# Clinical effects of integrated Chinese medicine therapy for postpartum pelvic floor dysfunction: a prospective patient-preference cohort study

**DOI:** 10.3389/fmed.2026.1782551

**Published:** 2026-04-02

**Authors:** Qingge Guo, Xiaowen Ma, Gaofeng Yang, Jiqin Yao, Liping Ni, Yaqin Qi, Suju Liu

**Affiliations:** 1 Department of Traditional Chinese Medicine, Hangzhou Women’s Hospital, Hangzhou, Zhejiang, China; 2 Department of Thyroid and Breast Surgery, Hangzhou Women’s Hospital, Hangzhou, Zhejiang, China; 3 Department of General Surgery, The Third Affiliated Hospital of Chongqing Medical University, Chongqing, China; 4 Department of Ultrasound, Hangzhou Women’s Hospital, Hangzhou, China; 5 Outpatient Care Unit, Hangzhou Women’s Hospital, Hangzhou, China; 6 Department of Gynecology, Hangzhou Women’s Hospital, Hangzhou, China

**Keywords:** acupoint stimulation, Dabu-Yuanjian Tisheng formula, Integrated Chinese medicine therapy, pelvic floor rehabilitation, postpartum pelvic floor dysfunction

## Abstract

**Objective:**

To evaluate the therapeutic efficacy of Dabu-Yuanjian Tisheng formula combined with acupoint stimulation for postpartum pelvic floor dysfunction (PFD) compared with single-modality and standard rehabilitation.

**Methods:**

This prospective, patient-preference observational study enrolled 212 postpartum women diagnosed with PFD, who chose one of four management options after standardized counseling. Group A received Dabu-Yuanjian Tisheng formula alone, Group B acupoint stimulation alone, Group C the combined therapy, and Group D standard electrical stimulation with biofeedback. Quota sampling targeted 53 participants per group. The 8-week intervention evaluated changes in POP-Q staging, urinary incontinence symptoms (ICIQ-SF), vaginal dynamic pressure, and traditional Chinese medicine (TCM) syndrome scores; pelvic floor muscle strength (Modified Oxford Scale) was assessed to characterize severity. Between-group comparisons used ANCOVA for continuous variables and proportional-odds models for ordinal outcomes, adjusted for prespecified covariates and baseline values. Missing data were handled via multiple imputation.

**Results:**

A total of 212 participants (53 per group) were analyzed. Baseline characteristics were generally comparable. All groups showed significant pre- to post-treatment improvement across major outcomes (all *p* < 0.00001). After adjustment, selecting the combined therapy (Group C) was associated with the most favorable improvements across measured outcomes. Vaginal dynamic pressure improved more in Group C than in Group A (mean + 3.59, *p* < 0.00001) and Group B (+ 2.21, *p* = 0.0340); Group D improved more than Group A (+ 2.30, *p* = 0.0277). For bladder neck–symphysis distance (BSD), Group C improved more than Group A (+ 0.172, *p* = 0.00209) and Group B (+ 0.246, *p* < 0.00001). Reductions in ICIQ-SF and TCM scores showed similar patterns, with Group C achieving the largest gains (all *p* < 0.00001). POP-Q staging distribution differed significantly among groups (*p* < 0.00001), favoring Group C. Sensitivity analyses confirmed robustness, with > 97% power for detecting clinically relevant differences.

**Conclusion:**

In this patient-preference cohort, the choice of Dabu-Yuanjian Tisheng formula combined with acupoint stimulation was associated with greater improvements in postpartum PFD outcomes compared to single-modality or standard rehabilitation. However, because treatment allocation was non-randomized and residual confounding cannot be excluded, these findings should be interpreted as supportive comparative evidence rather than definitive proof of superiority.

## 1 Introduction

Pelvic floor dysfunction (PFD) in women encompasses a spectrum of disorders resulting from damage, degeneration, or aging of pelvic floor tissues, leading to impaired function of related organs (1). Common risk factors include obstetric and gynecologic history (e.g., pregnancy, vaginal delivery, pelvic surgery, menopause) as well as obesity and chronic straining (2, 3). PFD represents a major public health concern, substantially affecting women’s physical and psychological wellbeing and overall quality of life (4).

Accumulating clinical evidence indicates that pregnancy and childbirth exert profound impacts on pelvic floor integrity and function (5). Epidemiological studies in China have shown that approximately 40% of women of childbearing age experience some degree of pelvic floor dysfunction (6). A retrospective analysis of stress urinary incontinence cases reported that 40.69% of patients developed symptoms within 12 months postpartum (7), highlighting this period as a peak incidence window. Under the current multiple-child policy, women may experience more frequent pregnancies and deliveries, further increasing the risk of postpartum pelvic floor dysfunction. Therefore, comprehensive postpartum rehabilitation strategies—including TCM, acupuncture, and pelvic floor muscle training—are increasingly recommended. These interventions have been shown to enhance pelvic floor muscle strength and reduce the incidence of urinary incontinence and organ prolapse, as consistently demonstrated by both domestic and international studies (8, 9).

Globally, postpartum pelvic floor rehabilitation primarily centers on Pelvic Floor Muscle Training (PFMT) and biofeedback-assisted electrical stimulation as frontline strategies (10). While effective, individual response variability and long-term adherence remain significant clinical hurdles in Western and Asian populations alike (11). Currently, there is a lack of high-quality prospective evidence exploring integrative protocols that combine these physical modalities with traditional adjunctive therapies to optimize recovery trajectories. Our study addresses this evidence gap by evaluating a standardized Chinese medicine formula and acupoint stimulation within a multi-modal framework, providing a comparative perspective that may broaden international options for personalized pelvic floor care. Currently, the standard clinical treatment for postpartum pelvic floor dysfunction mainly relies on pelvic floor electrical stimulation combined with biofeedback therapy, which focuses on strengthening pelvic floor musculature. However, many postpartum women experience general physical weakness and find it difficult to visit hospitals frequently or to master correct pelvic floor muscle contraction techniques, thereby limiting rehabilitation outcomes. TCM emphasizes holistic balance and the harmony between the human body and its environment (12) 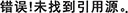 Guided by these principles, TCM practitioners often employ integrated approaches combining herbal formulations with acupuncture to promote postpartum recovery. Recent domestic studies have revealed that deficiency syndromes—such as Qi deficiency, Qi sinking, and insufficiency of Qi—account for approximately 73% of postpartum pelvic floor dysfunction cases, which are consistent with the indications for treatment using traditional Chinese herbal decoctions (13). In this context, the present study investigates the therapeutic effects of Dabu-Yuanjian Tisheng formula combined with acupoint stimulation in the management of postpartum pelvic floor dysfunction, aiming to evaluate its clinical efficacy and provide further insights into the underlying mechanisms.

## 2 Materials and methods

This was a prospective, patient-preference observational study conducted at Hangzhou Women’s Hospital (Hangzhou, China). The protocol was approved by the Ethics Committee of Hangzhou Women’s Hospital (Approval No. [2018] Medical Ethics Review (008)-01). All participants provided written informed consent prior to enrollment. The study period was January 1, 2019 through December 31, 2022. Women attending the Department of Traditional Chinese Medicine (Gynecology) who were diagnosed with pelvic floor dysfunction (PFD) were consecutively screened for eligibility. After standardized counseling on all available management options, eligible participants selected their preferred strategy and were assigned accordingly. To ensure adequate precision for between-group comparisons and to balance clinical workload, quota sampling was prospectively implemented with a target number per group; once a group reached capacity, subsequent eligible participants could choose among the remaining options or decline participation. General demographics and disease-related information were recorded at baseline prior to treatment initiation. This study is reported in accordance with the STROBE statement for observational studies.

While a randomized controlled trial (RCT) is the rigorous gold standard for establishing causal efficacy, a patient-preference observational design was deliberately chosen for this study to maximize ecological validity. In our real-world clinical setting, postpartum women often hold strong cultural and personal preferences regarding the use of Traditional Chinese Medicine (TCM) versus device-based therapies. A preference-based design respects patient autonomy, maximizes treatment adherence, and accurately reflects routine clinical practice, although we explicitly acknowledge that it precludes definitive causal inference due to inherent selection bias.

### 2.1 Diagnostic definition of PFD

PFD was defined as a spectrum of disorders due to functional abnormalities of pelvic floor muscles, fascia, and their neural control, resulting in impaired support of pelvic organs and/or lower urinary tract, bowel, or sexual function. A clinical diagnosis required typical symptoms together with objective confirmation of functional or anatomical abnormalities (4, 10).

### 2.2 Required domains and qualifying findings

Typical symptoms: lower urinary tract symptoms (frequency, urgency urinary incontinence, stress urinary incontinence, voiding difficulty); bowel symptoms (constipation, incomplete evacuation, need for digital assistance); prolapse symptoms (vaginal/perineal bulge or pressure, foreign-body sensation); sexual dysfunction (dyspareunia, decreased arousal/orgasm); chronic pelvic floor or perineal pain.

Physical examination: pelvic organ prolapse by Pelvic Organ Prolapse Quantification (POP-Q) staging (Stage ≥ II for prolapse-type PFD); abnormal pelvic floor muscle tone (hypertonia or hypotonia) or reduced strength [Modified Oxford Scale (14) ≤ 3]; impaired coordination or unilateral contraction deficit.

Functional testing: surface electromyography (sEMG) abnormalities in contraction peak or relaxation phase; abnormal vaginal or anal manometry indicating reduced sphincter function; urodynamics consistent with voiding dysfunction or urinary incontinence (stress, urgency, or mixed); anal manometry showing reduced resting/squeeze tone or absent reflexes.

Imaging/electrophysiology (supportive): perineal or transvaginal ultrasound showing descent of pelvic floor structures, increased urethral mobility, or organ prolapse; dynamic MRI indicating multi-compartment prolapse, levator ani avulsion, or fascial defects.

### 2.3 Inclusion criteria

1. Age 18–45 years; 2. Singleton live birth; 3. Primiparous; 4. Spontaneous vaginal delivery; 5. Postpartum window; 6. 42 days to 2 months at enrollment; 7. Lochia resolved; 8. Perineal or cesarean incision healed; 9. Confirmed PFD as defined above with at least one of: POP-Q Stage I–II pelvic organ prolapse; pelvic floor muscle strength Modified Oxford Scale ≤ 3; vaginal dynamic pressure below age-matched postpartum reference values; 10. Baseline standardization: received unified postpartum pelvic floor rehabilitation education and performed self-exercise ≥ 1 week; 11. Willing to continue per protocol.

Adherence and follow-up: able to complete 8-week intervention and scheduled evaluations; provided written informed consent; Safety: no known contraindications or allergy to electrical stimulation, acupoint stimulation, or the study decoction.

### 2.4 Exclusion criteria

1. POP-Q Stage III–IV or need for imminent surgical intervention or pessary. 2. Unhealed acute perineal trauma (III/IV perineal tear), active wound infection, unresolved lochia. 3. Active urogenital infection (acute vaginitis, pelvic inflammatory disease, urinary tract infection) or uncontrolled conditions causing sustained intra-abdominal pressure (e.g., severe constipation, chronic cough). 4. Prior pelvic floor repair/vaginal surgery or neurological disorders affecting pelvic floor function (e.g., spinal cord disease, multiple sclerosis, stroke sequelae). 5.Known connective tissue disease, severe obesity (BMI ≥ 35), or heavy manual labor causing progressive mechanical descent unlikely to improve with conservative therapy. 6. Contraindications to electrostimulation/biofeedback: pacemaker/implantable defibrillator, uncontrolled epilepsy, mucosal ulceration/bleeding at probe site, current or suspected pregnancy. 7. Contraindications to acupoint stimulation: bleeding diathesis or anticoagulation precluding needling; local skin infection/ulceration; severe allergy to acupuncture/patching. 8. Contraindications to the Dabu-Yuanjian Tisheng formula (Group A/C): allergy to any component [Angelica sinensis, Rehmannia glutinosa (prepared), Cornus officinalis, Lycium barbarum, Eucommia ulmoides, fried Codonopsis pilosula, Dioscorea opposita, Astragalus membranaceus, Glycyrrhiza uralensis, Cimicifuga heracleifolia, Alpinia oxyphylla, Euryale ferox], severe hepatic/renal dysfunction, or uncontrolled hypertension/diabetes/thyroid disease requiring changes to background therapy. 9. Lactation considerations: strong personal objection to herbal intake during breastfeeding (eligible for Groups B/D only, not A/C). 10. Concomitant therapies within 4 weeks before enrollment: local/systemic estrogen or hormonal contraception; pessary use; other systemic pelvic floor therapies (acupuncture, tuina, herbal medicine, intensive electrostimulation) without adequate washout. 11. Severe cardiovascular/cerebrovascular events within 3 months (myocardial infarction, stroke), active malignancy, or psychiatric disorders precluding cooperation with evaluation.

### 2.5 Allocation (patient preference)

After eligibility confirmation and standardized counseling on all available management options, participants selected their preferred treatment strategy and were categorized into one of four groups (A–D). To ensure adequate precision for between-group comparisons and to balance clinical workload, quota sampling was prospectively implemented with a target of 53 participants per group (total *N* = 212). Once a group reached capacity, subsequent eligible participants could choose among the remaining options or decline participation. Because treatment selection was based on patient preference, no randomization was performed. Given the nature of the interventions, the study was open-label and no masking was applied.

### 2.6 Interventions

#### 2.6.1 Group A: traditional Chinese medicine decoction

Group A received the Dabu Yuanjian Tisheng traditional Chinese medicine decoction starting at least 42 days postpartum for an 8 week course. The daily formula consisted of Angelica sinensis 10 g, prepared Rehmannia 12 g, Cornus officinalis 10 g, Lycium barbarum 10 g, Eucommia ulmoides 10 g, fried Codonopsis pilosula 12 g, Dioscorea opposita 15 g, Astragalus membranaceus 20 g, Glycyrrhiza uralensis 6 g, Cimicifuga heracleifolia 6 g, Alpinia oxyphylla 10 g, and Euryale ferox 15 g. For preparation, the herbs were soaked in 600–800 mL of water for 30 min and then decocted for 30–40 min to yield approximately 300 mL, which was taken warm in two divided doses of about 150 mL each morning and evening.

To ensure the standardization of the herbal preparation, all raw medicinal materials were sourced from the central pharmacy of Hangzhou Women’s Hospital, strictly adhering to the quality standards of the Pharmacopoeia of the People’s Republic of China (2020 Edition). The decoction process was centralized and automated using a standardized extraction and vacuum-packaging system (Model: YZ-200, or equivalent) to maintain batch-to-batch consistency in concentration and chemical potency. Each 200 mL dose was vacuum-sealed and assigned a batch tracking number to ensure the quality control of herbal compounds throughout the study duration.

Physician guided modifications were permitted according to prespecified symptom patterns, such as increasing Astragalus or adding herbs for specific complaints, and all changes were documented with the date, indication, herbs added or removed, dosage, and duration. Quality control included recording the herb supplier, batch number, and receipt inspection information. Adherence was assessed using a daily dosing diary supplemented by weekly return of empty packaging or photo verification, calculated as doses taken divided by planned doses times 100, with protocol adherence defined as at least 80 percent. Safety was monitored weekly by recording adverse reactions, and treatment was discontinued in the event of suspected allergy or clinically significant liver or renal function abnormalities.

#### 2.6.2 Group B: acupoint stimulation using suction type low frequency therapy

Group B received acupoint stimulation using a suction type low frequency therapy device in mixed mode. The acupoints included bilateral Zigong EX CA1, Guanyuan CV4, bilateral Sanyinjiao SP6, and bilateral Shenshu BL23. Zigong EX CA1 was located 3 cun lateral to CV3 on the lower abdomen, Guanyuan CV4 was located on the anterior midline 3 cun inferior to the umbilicus, Sanyinjiao SP6 was located 3 cun proximal to the medial malleolus posterior to the medial tibial border, and Shenshu BL23 was located 1.5 cun lateral to the lower border of the L2 spinous process.

Treatment was administered twice weekly for 8 weeks, with each session lasting 30 min and suction pressure set at 20 kPa, yielding 16 planned sessions. Stimulation intensity was increased gradually from zero to a strong but comfortable level without pain, and the final intensity level or device scale value was recorded at each session. All procedures were delivered by licensed acupuncturists or trained TCM nurses or therapists with verified competency in acupoint localization and emergency management. Session fidelity was ensured using a standardized checklist documenting patient position, acupoint confirmation, suction pressure, total treatment time, recorded intensity, and any adverse events. Adherence was calculated as completed sessions divided by planned sessions times 100, and per protocol adherence was defined as completing at least 75 percent of sessions.

Group C: Combination therapy

Participants received both Group A decoction (same formula/preparation/dose) and Group B acupoint stimulation (same acupoints/parameters/schedule) for 8 weeks. Adherence: calculate separately for decoction and sessions; define combination adherence as meeting both thresholds.

#### 2.6.3 Group D: standard pelvic floor rehabilitation

Participants received pelvic floor neuromuscular electrical stimulation followed by EMG biofeedback training using the PHENIX USB4 system (ELECTRONIC CONCEPT LIGNON INNOVATION, France) with a vaginal probe. Each session lasted 30 min and was delivered twice weekly for 8 weeks (16 sessions). The NMES phase used the manufacturer preset program for pelvic floor muscle strengthening for stress urinary incontinence, with biphasic symmetrical pulses, frequency 50 Hz, pulse width 300 ms, duty cycle 5 s on and 10 s off, with 2 s ramp up and 2 s ramp down. Stimulation intensity was titrated from 0 to the highest comfortable level that elicited a clear pelvic floor contraction without pain, and the peak intensity was recorded for each session. Immediately after NMES, participants completed a 10 min EMG biofeedback training block consisting of 10 repetitions of sustained contractions held for 6–8 s with 10 s rest, followed by 10 rapid contractions, repeated for two cycles as tolerated. All sessions were delivered by trained pelvic floor rehabilitation therapists, and session fidelity was monitored using a standardized checklist recording program type, frequency, pulse width, duty cycle, ramp time, total NMES duration, biofeedback duration, and peak stimulation intensity.

### 2.7 Outcome assessments and measurement procedures

POP-Q staging: The POP-Q system was used to quantify anterior (Aa, Ba), apical (C, D), and posterior (Ap, Bp) compartment descent with three reference lines. Staging criteria: Stage I (< –1 cm relative to hymen), Stage II (–1 to + 1 cm), Stage III (> + 1 cm to < TVL – 2 cm), and Stage IV (≥ TVL – 2 cm) (15). Vaginal dynamic pressure: Vaginal dynamic pressure was measured using the PHENIX USB4 system (16). A pressure balloon covered with an oil-free condom was lubricated with paraffin oil and inserted gently into the mid-vagina. Air was injected to ensure full contact with the vaginal wall. Participants were instructed to perform maximal pelvic floor contraction, and the peak pressure was recorded. Normal reference values are 80–150 cmH_2_O; lower values suggest impaired urinary control and sexual function.

Pelvic floor ultrasound protocol—bladder neck–symphysis distance BSD score: Ultrasound examinations were performed on a GE Voluson E8 with an RE-6-10 3D volume probe (6–10 MHz). The 2D scan angle was 70° and 3D angle 85° (17). After rectal and bladder emptying (residual < 50 mL), participants were examined in lithotomy with hips flexed and abducted. A sheathed probe with sterile coupling gel was positioned between the labia majora to acquire mid-sagittal images using the inferior-posterior edge of the pubic symphysis as the reference. During maximal Valsalva, pelvic organ positions were measured. The prespecified primary ultrasound indicator was the BSD, defined as the vertical distance from the bladder neck to the inferior edge of the pubic symphysis. A position at or above the reference line is normal; descent below the line indicates bladder prolapse. Each measurement was obtained three times and averaged.

Urinary incontinence symptoms: The International Consultation on Incontinence Questionnaire–Short Form (ICIQ-SF) was administered at baseline and after 8 weeks to assess the frequency/volume of urinary leakage and impact on quality of life (total score 0–21; higher scores indicate more severe incontinence) (18). Change in ICIQ-SF (ΔICIQ-SF) was calculated as baseline minus post-treatment score; higher positive values indicate greater improvement.

TCM syndrome quantitative scoring: TCM syndrome scores were assessed as a secondary symptom-based outcome within the traditional Chinese medicine framework. According to the Guiding Principles for Clinical Research on New Chinese Medicines (19), TCM syndrome scores were collected at baseline and post-treatment. Core items included fatigue/reluctance to speak, pale complexion, limb weakness, and frequent urination. Severity was graded as mild (2 points), moderate (4 points), or severe (6 points), with summed total scores reflecting overall syndrome burden Given its limited international use and the lack of broad validation specifically for postpartum pelvic floor dysfunction, this measure was interpreted as a supportive secondary outcome rather than a primary efficacy endpoint.

#### 2.7.1 Severity grading and integrated classification

Pelvic floor muscle strength was assessed by digital vaginal examination using the Modified Oxford Scale (0–5): 0, no palpable contraction; 1, flicker; 2, weak and brief; 3, moderate, sustained for 2–4 s; 4, good, resisting counter-pressure for > 4 s; 5, strong, circumferential contraction resisting strong counter-pressure for > 5 s. Scores 0–2 indicate marked weakness, 3–4 moderate dysfunction, and 5 normal/good function. As a subjective measure, this grading was used solely to characterize baseline pelvic floor muscle strength and clinical severity; it was not a prespecified comparative endpoint.

Anatomical severity followed POP-Q staging: Stage 0, no prolapse; Stage I, most distal point > 1 cm above hymen; Stage II, within ± 1 cm; Stage III, > 1 cm below hymen but < TVL - 2 cm; Stage IV, near or complete vaginal eversion. POP-Q Stage II or higher was considered clinically significant prolapse requiring intervention. Functional status was graded by the Modified Oxford Scale (0–5) as above. An integrated clinical classification guided care: mild (early)—mild symptoms, POP-Q I–II, Oxford ≥ 3, no major structural damage (conservative rehabilitation appropriate); moderate (progressive)—prominent symptoms (e.g., PFDI > 33), POP-Q II–III, Oxford ≤ 3, possible mild urinary/defecatory dysfunction (structured rehabilitation ± support devices); severe (structural)—POP-Q III–IV and/or Oxford ≤ 2 with significant incontinence or externalized prolapse (often requires reconstructive surgery) (14).

Concomitant care and restrictions: Participants received standardized postpartum education and continued prescribed self-exercises. Per exclusion criteria, recent use of local/systemic estrogen or hormonal contraception, pessary placement, or other systemic pelvic floor therapies required washout before enrollment. During the intervention period, additional therapies for pelvic floor dysfunction were discouraged unless medically necessary.

### 2.8 Follow-up and data collection

Participants were followed according to group allocation and clinical schedules for an 8-week intervention period. Baseline demographics, obstetric history, and potential confounders (age, BMI, hemoglobin, education level calcium supplementation, Postpartum days, Weight change postpartum compared to pre pregnancy, breastfeeding status, frequency of self-training) were recorded. At baseline and follow-up, assessments included symptoms, physical examination, POP-Q staging, pelvic floor muscle strength, vaginal dynamic pressure, pelvic floor ultrasound (including BSD), ICIQ-SF, and TCM syndrome scores. Safety monitoring included review of medical history, liver/renal function, concomitant medications, and allergies to acupuncture/herbs/electrical stimulation. All procedures followed departmental standard operating protocols, with efforts to maintain the same equipment and operator for serial measurements wherever feasible.

### 2.9 Statistical analysis method

#### 2.9.1 Patient-preference allocation and baseline balance assessment

Eligible participants selected their preferred management strategy after standardized counseling and were categorized into four groups. Quota sampling was prospectively implemented with a target of 53 participants per group (total *N* = 212). Because allocation was not randomized, baseline comparability was assessed using prespecified clinically relevant variables, including: (1) quantified TCM syndrome total score, (2) POP-Q stage, (3) pelvic floor muscle strength (Modified Oxford Scale grading), and (4) other baseline clinical descriptor. Group-level balance was evaluated using standardized mean differences (SMDs) and descriptive summary tables. Variables showing meaningful imbalance as well as clinically important baseline factors identified *a priori*, were accounted for in adjusted analyses as prespecified covariates.

To clarify the presumed confounding structure of this patient-preference cohort, we constructed a directed acyclic graph (Supplementary Figure 3), which informed our interpretation of measured confounding, unmeasured bias, and residual causal uncertainty.

#### 2.9.2 Measurement standardization

To minimize measurement variability, assessments were performed using the same operator and the same device whenever feasible. Bladder filling and respiratory cooperation were standardized and documented in a written standard operating procedure, including Valsalva maneuver standardization (instruction script, practice trials, acceptance criteria, and discard rules for suboptimal attempts).

#### 2.9.3 Intervention quality control

For the Dabu-Yuanjian Tisheng formula, the base prescription and administration procedures were standardized across participants, including (1) daily dose (grams/day of crude herbs), (2) decoction procedure (water volume, soaking time, decoction time, and final volume), and (3) timing of administration. Herb source, batch information, and quality documentation were recorded. A breastfeeding education card and a management card for common discomfort/adverse reactions were provided and documented as part of quality control. Standard operating procedures were also applied for acupoint stimulation and electrical stimulation/biofeedback sessions to ensure consistency in device settings, session duration, and delivery.

#### 2.9.4 Outcome assessment and data collection

Given the patient-preference nature of treatment allocation and the practical constraints of the clinical setting, the study was conducted as open-label. To reduce assessment bias, a strict separation was maintained between the treatment team and the outcome assessors. Clinical evaluations, including the Modified Oxford Scale and POP-Q staging, as well as ultrasound BSD measurements, were performed by experienced physicians and sonographers who were not involved in treatment delivery and were blinded to group allocation whenever feasible. Participants were instructed not to disclose their assigned intervention during follow-up assessments. Assessors followed standardized measurement protocols, and questionnaire-based outcomes were completed independently by participants whenever feasible and were administered or collected by staff not involved in intervention delivery. Data were recorded using prespecified case report forms, and treatment groups were coded in the analysis dataset prior to the primary statistical analyses.

#### 2.9.5 Outcomes

Pre-specified primary outcomes were change from baseline to the end of treatment in: POP-Q stage (ordinal), urinary incontinence symptoms (ICIQ-UI-SF score), and vaginal dynamic pressure (manometry). The primary POP-Q endpoint was the change in POP-Q stage from baseline to week 8 (end of treatment); improvement was defined as a decrease of at least one stage. Secondary outcomes included the quantified TCM syndrome score and BSD score. Safety endpoints included adverse events related to the interventions and breastfeeding.

#### 2.9.6 Sample size

The sample size targeted detection of a moderate effect size for a four-arm comparison using one-way ANOVA. Assuming Cohen’s *f* = 0.25, two-sided α = 0.05, and power = 80%, the required total was *N* = 212 (53 per arm). To manage attrition, we planned for 15% drop-out and prepared a 10% reserve list (≈21 candidates). If attrition exceeded 15%, reserve candidates were to be activated. The calculation was performed in R using pwr.anova.test (*k* = 4, *f* = 0.25, sig.level = 0.05, power = 0.80).

#### 2.9.7 Statistical analysis

Because allocation was based on patient preference rather than randomization, analyses followed an as-allocated (as-selected) principle, with all participants analyzed in their chosen group. A per-protocol (adherence-based) analysis was conducted as supportive. To improve interpretability, analyses were organized hierarchically as primary analyses, secondary/supportive analyses, and sensitivity analyses. Continuous variables were summarized as mean (SD) or median (IQR), as appropriate, and categorical variables as counts and percentages. Model assumptions, including normality and homoscedasticity, were assessed using diagnostic plots and formal tests where relevant.

#### 2.9.8 Primary analysis

Primary analyses focused on adjusted between-group comparisons at the end of treatment while accounting for baseline values and prespecified covariates. For continuous primary outcomes, analysis of covariance (ANCOVA) models were used, with treatment group as the main factor and the baseline value of the corresponding outcome plus the following prespecified covariates entered as adjustment variables: age, body mass index, hemoglobin, education level, calcium supplementation, postpartum days, postpartum weight change relative to pre-pregnancy, breastfeeding status, and frequency of self-training.

For the ordinal primary outcome, POP-Q stage, proportional odds regression models were fitted with adjustment for the same covariates. The proportional odds assumption and overall model fit were assessed using Brant-type tests and other relevant diagnostic procedures.

For all primary outcomes, results are reported as adjusted effect estimates with 95% confidence intervals and two-sided *p*-values. Continuous outcomes are presented as adjusted mean differences, whereas ordinal outcomes are presented as adjusted odds ratios. Omnibus group tests, when reported, were used as supportive global tests and were not emphasized over effect estimates.

If assumptions for the primary continuous-outcome models were not adequately satisfied, alternative approaches, including rank-based ANCOVA or generalized linear models with appropriate link functions, were examined as robustness checks.

#### 2.9.9 Secondary outcomes

The TCM syndrome score and BSD score (continuous) used the same ANCOVA framework. These analyses were interpreted as supportive rather than definitive efficacy analyses.

Repeated-measures or longitudinal modeling approaches, when applied, were considered supportive analyses intended to characterize temporal patterns than replace the primary endpoint models.

For patient-reported and physiologic outcomes, clinical relevance was considered alongside statistical significance. Where established minimal clinically important differences were available, they were used to aid interpretation. For outcomes without well-established MCIDs, results were interpreted cautiously on the basis of effect magnitude, confidence intervals, and consistency across related endpoints.

#### 2.9.10 Multiplicity

To limit false-positive findings across multiple endpoints, the family-wise error rate for the prespecified primary outcomes was controlled using the Holm procedure with a two-sided α of 0.05. Within-group pre–post comparisons were also adjusted using the Holm procedure where applicable. For between-group pairwise comparisons in the four-group setting, multiplicity was addressed using Tukey-adjusted tests when appropriate. Secondary analyses were interpreted as supportive rather than definitive, and, where multiple secondary comparisons were summarized, the Benjamini–Hochberg false discovery rate (FDR) procedure was applied as appropriate.

### 2.10 Supplementary methods propensity score based sensitivity analyses

To evaluate the robustness of the primary findings to confounding and selection bias arising from patient preference allocation, we conducted propensity score based sensitivity analyses on the completed imputed dataset. A generalized propensity score for the four treatment strategies from Group A to Group D was estimated using multinomial logistic regression including prespecified baseline covariates measured prior to treatment, namely age, educational attainment, body mass index, postpartum days, number of pregnancies, hemoglobin, calcium supplementation, postpartum weight change compared with pre pregnancy, lactation status, baseline pelvic floor muscle strength type, baseline vaginal dynamic pressure, baseline urinary incontinence questionnaire score, baseline BSD score, baseline TCM syndrome score, and baseline POP Q staging.

Using the estimated generalized propensity score, we constructed stabilized inverse probability of treatment weights targeting the average treatment effect and generalized overlap weights targeting the overlap population. To reduce the influence of extreme weights, stabilized inverse probability of treatment weights were trimmed at the first and ninety ninth percentiles. Weight diagnostics were summarized using distributional summaries as shown in Supplementary Table 1.

Covariate balance before and after weighting was assessed using the maximum absolute standardized mean difference across all pairwise group contrasts for each baseline covariate, with values below 0.10 considered acceptable; detailed balance statistics are presented in Supplementary Tables 2, 3 and Supplementary Figure 1.

Treatment effects were re estimated using weighted only marginal models including group indicators only and doubly robust weighted models that additionally adjusted for the baseline value of the corresponding outcome and the prespecified covariates used in the primary analysis. Continuous outcomes were analyzed using weighted linear regression with heteroskedasticity robust standard errors and are reported as adjusted mean differences with 95 percent confidence intervals in Supplementary Table 4. POP Q staging at follow up was analyzed as a binary endpoint using weighted logistic regression with robust standard errors and is reported as odds ratios with 95 percent confidence intervals in Supplementary Table 5. All comparisons were expressed relative to Group D.

### 2.11 Missing data

Several outcomes exhibited missingness, including post-treatment vaginal dynamic pressure, post-treatment TCM syndrome score, post-treatment POP-Q stage, post-treatment urinary incontinence symptoms (ICIQ-UI-SF), and post-treatment BSD, with an overall missing rate of approximately 11–13%. We explored the missing-data mechanism using Little’s MCAR test for numeric variables (custom implementation following the BaylorEdPsych framework). The test was non-significant (e.g., χ^2^ = 3183.173, df = 3263, *p* = 0.838), which did not provide evidence against MCAR; however, given the limitations of statistical tests for missingness mechanisms, we treated the missingness as at least missing at random (MAR) and performed multiple imputation accordingly.

Because missingness was moderate, we performed Multiple Imputation (MI) in R using the mice package (*m* = 20). Continuous variables (e.g., hemoglobin, number of pregnancies, postpartum vs. pre-pregnancy weight change, vaginal dynamic pressure, TCM score, BSD, and ICIQ-UI-SF score) were imputed using predictive mean matching (PMM). Binary variables (e.g., lactation, calcium tablet intake) were imputed using logistic regression (logreg). Nominal variables (e.g., education level) were imputed using multinomial logistic regression (polyreg). Ordinal variables (e.g., Modified Oxford Scale grading, POP-Q stage) were imputed using proportional odds logistic regression (polr). Variables were pre-typed as factor/ordered factor as appropriate. Treatment group (patient-preference allocation) and other fixed design variables were not imputed; treatment group was included as a predictor in the imputation models together with baseline covariates and auxiliary variables plausibly related to missingness and outcomes to improve efficiency and reduce bias. Pooled estimates were obtained using Rubin’s rules.

### 2.12 Software and reproducibility

All analyses were conducted in R,^1^ using base stats, pwr (sample size planning), mice (multiple imputation), and modeling packages for ANCOVA and ordinal regression. Scripts, random seeds, and session information are available upon request to facilitate reproducibility.

### 2.13 Multiple imputation and sensitivity analyses

**FIGURE 1:**
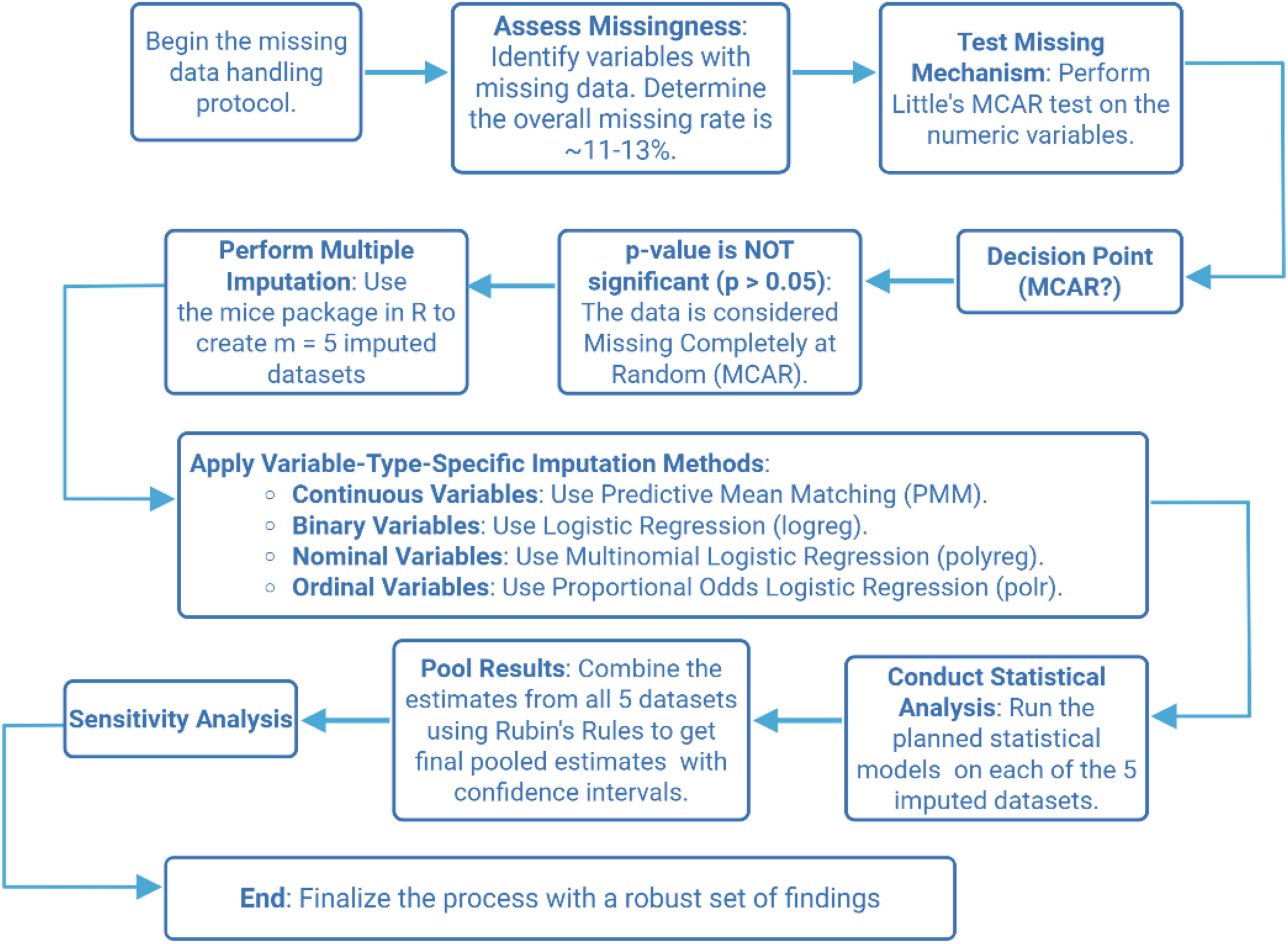
Data missing processing flowchart.

All imputations were performed using the mice package and pooled using Rubin’s rules. Sensitivity analyses were conducted on both complete-case and MI-pooled datasets. For each outcome, we applied the same regression specification (identical predictors and covariate set) across datasets. The direction and magnitude of estimates were highly consistent between complete-case and MI-pooled analyses, supporting the robustness of the findings to the handling of missing data (Figure 1).

### 2.14 Imputation diagnostics and plausibility check

**FIGURE 2:**
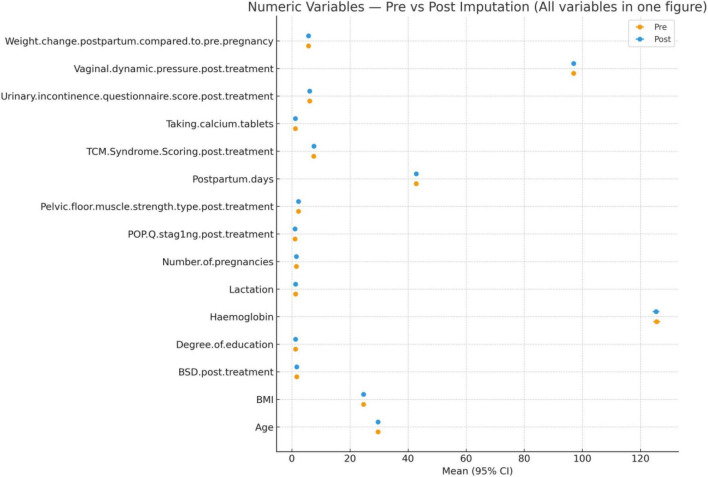
Imputation diagnostics and plausibility check.

To assess the plausibility and stability of the imputed data, we compared pre- and post-imputation means with 95% confidence intervals for all continuous variables. Post-imputation estimates closely matched the original values, and the CIs largely overlapped, indicating that the imputation procedure preserved the original data distribution without introducing systematic bias. No variable showed a clinically or statistically meaningful shift after imputation; minor CI tightening in a few variables reflected the larger effective sample size rather than a change in central tendency. Overall, these findings demonstrate that the imputation process maintained the integrity of the original data and supports the validity of subsequent analyses using the imputed dataset (Figure 2).

## 3 Results

To ensure clarity and consistency in our outcome reporting, the direction of clinical improvement for each metric was predefined. For vaginal dynamic pressure and BSD score, a positive change or higher score (post-treatment > pre-treatment) indicates functional and anatomical improvement. Conversely, for the ICIQ-UI-SF and TCM syndrome score, a reduction in the raw score (post-treatment < pre-treatment) signifies symptom alleviation. All between-group effect estimates are reported with 95% confidence intervals (CIs) derived from our adjusted models.

### 3.1 Baseline characteristics

A total of 212 patients were included in the final analysis and were allocated into four groups (Groups A–D) based on patient preference with prospective quota sampling (53 patients per group).

Baseline demographic and clinical characteristics are summarized in Table 1. Overall, the groups were broadly comparable at baseline. The mean age ranged from 29.47 to 29.53 years, and mean BMI ranged from 24.14 to 25.07 kg/m^2^. Other continuous variables, including hemoglobin level, postpartum days, number of pregnancies, and postpartum weight change, were similar across groups with no statistically significant differences (all *p*-values > 0.05).

**TABLE 1:** Baseline characteristics of the study population.

Variable	Level	Group A	Group B	Group C	Group D	*p*
*n*		53	53	53	53	0.779
Age, years		29.47 (4.07)	30.15 (3.96)	29.45 (4.10)	29.53 (4.03)	
BMI, kg/m^2^		24.14 (1.84)	25.07 (1.99)	24.68 (1.68)	24.78 (1.93)	0.079
Hemoglobin g/L		126.30 (8.18)	124.28 (9.52)	126.25 (8.17)	124.57 (8.88)	0.491
Postpartum days		42.91 (1.99)	42.75 (2.02)	42.68 (1.83)	42.92 (1.81)	0.894
Number of pregnancies		1.57 (0.54)	1.55 (0.57)	1.55 (0.61)	1.58 (0.57)	0.984
Weight change postpartum compared to prepregnancy		5.81 (2.26)	5.92 (2.38)	6.02 (2.32)	5.15 (1.89)	0.177
Baseline vaginal dynamic pressure		79.02 (3.77)	78.91 (3.59)	77.66 (4.18)	77.53 (4.48)	0.106
Baseline urinary incontinence questionnaire score		11.23 (1.22)	11.13 (1.26)	11.25 (1.19)	11.43 (0.93)	0.594
Baseline BSD		1.24 (0.28)	1.14 (0.27)	1.10 (0.28)	1.09 (0.35)	0.04
Baseline TCM syndrome scoring		15.77 (0.72)	15.70 (0.82)	15.83 (0.61)	15.77 (0.72)	0.828
Degree.of.education (%)	1	34 (64.2)	39 (73.6)	42 (79.2)	38 (71.7)	0.38
	2	19 (35.8)	14 (26.4)	11 (20.8)	15 (28.3)	
Taking.calcium.tablets (%)	1	41 (77.4)	47 (88.7)	40 (75.5)	40 (75.5)	0.272
	2	12 (22.6)	6 (11.3)	13 (24.5)	13 (24.5)	
Lactation (%)	1	36 (67.9)	40 (75.5)	38 (71.7)	40 (75.5)	0.791
	2	17 (32.1)	13 (24.5)	15 (28.3)	13 (24.5)	
Baseline POP.Q.stage (%)	1	39 (73.6)	35 (66.0)	31 (58.5)	30 (56.6)	0.247
	2	14 (26.4)	18 (34.0)	22 (41.5)	23 (43.4)	
Basline pelvic floor muscle strength type (%)	0	8 (15.1)	6 (11.3)	6 (11.3)	7 (13.2)	0.99
Basline POP.Q.stag1 (%)	1	30 (56.6)	31 (58.5)	29 (54.7)	31 (58.5)	
	2	15 (28.3)	16 (30.2)	18 (34.0)	15 (28.3)	

Regarding baseline clinical indicators prior to treatment, no significant between-group differences were observed in vaginal dynamic pressure, urinary incontinence questionnaire score, or TCM syndrome score (all *p*-values > 0.05). However, a modest but statistically significant difference was noted in the baseline bladder neck–symphysis distance (BSD.pre.treatment) (p = 0.04), with Group A showing a slightly higher mean value.

For categorical variables, distributions of education level, calcium tablet intake, lactation status, POP-Q stage, and pelvic floor muscle strength category were similar across groups at baseline (all *p*-values > 0.05).

Given the non-random, patient-preference allocation, baseline imbalances were anticipated. Therefore, subsequent between-group comparisons were performed using prespecified adjusted models that accounted for baseline covariates and baseline outcome values, including BSD where applicable.

### 3.2 Comparison of baseline balance between groups

Because treatment selection was based on patient preference rather than randomization, baseline balance across groups was evaluated descriptively using standardized mean differences (SMDs) for all baseline covariates and baseline outcome values. An SMD < 0.10 was prespecified to indicate negligible imbalance.

Overall balance. Across 25 baseline variables, 7 showed negligible imbalance (SMD < 0.10) and 18 showed meaningful imbalance (SMD ≥ 0.10) (Table 2). Notable imbalances were observed in clinically relevant measures (e.g., BMI, pelvic floor function indices, and POP-Q staging). These variables were therefore included as prespecified covariates in the adjusted outcome models to reduce confounding due to non-random allocation.

**TABLE 2:** Baseline balance summary across treatment groups before weighting.

Variable	Max.Diff.Un	Balance status
Age, years	0.1727	Not balanced, > 0.1
BMI, kg/m^2^	0.4978	Not balanced, > 0.1
Hemoglobin, g/L	0.2319	Not balanced, > 0.1
Postpartum days	0.1282	Not balanced, > 0.1
Number of pregnancies	0.0659	Balanced, < 0.1
Weight.change.postpartum.compared.to.pre.pregnancy	0.3909	Not balanced, > 0.1
Basline Vaginal dynamic pressure	0.3707	Not balanced, > 0.1
Baline urinary incontinence questionnaire score	0.261	Not balanced, > 0.1
Basline BSD	0.5045	Not balanced, > 0.1
Basline TCM syndrome scoring	0.1823	Not balanced, > 0.1
Degree of education (undergraduate and below)	0.1509	Not balanced, > 0.1
Taking calcium tablets (No)	0.1321	Not balanced, > 0.1
Lactation (No)	0.0755	Balanced, < 0.1
Basline POP.Q.stag.(II)	0.1698	Not balanced, > 0.1
Basline pelvic floor muscle strength type (0)	0.0377	Balanced, < 0.1
Basline pelvic floor muscle strength type (1)	0.0377	Balanced, < 0.1
Basline pelvic floor muscle strength type (2)	0.0566	Balanced, < 0.1

Model diagnostics. After covariate adjustment, model diagnostics (including residual checks and fit assessments) supported the adequacy of the analytical framework. The primary findings were materially unchanged in direction and magnitude across alternative specifications (e.g., complete-case vs. MI-pooled analyses), supporting robustness.

### 3.3 Within-group comparisons of efficacy before and after treatment

Model-based within-group analyses showed statistically significant changes from baseline to post-treatment across all study groups for all evaluated outcomes (all Holm-adjusted *p* < 0.00001; Supplementary Table 6). For vaginal dynamic pressure, all groups demonstrated improvement over the 8-week treatment period, with the largest adjusted change observed in Group C, followed by Groups D, B, and A. A similar pattern was observed for BSD, with all groups showing significant improvement and Group C exhibiting the greatest adjusted change.

For urinary incontinence symptoms assessed by the ICIQ-UI-SF, all groups showed significant improvement after treatment. The largest relative improvement was observed in Group C, followed by Group D, whereas Groups A and B showed smaller but still statistically significant changes. For POP-Q stage, significant within-group improvement was also observed in all groups, with the greatest model-based change seen in Group C. TCM syndrome scores decreased significantly in all groups, and the largest reduction was again observed in Group C.

Overall, all four interventions were associated with statistically significant within-group improvement over the 8-week study period. However, because postpartum pelvic floor function may also improve over time owing to natural recovery and concurrent self-care, these within-group findings should be interpreted descriptively. The primary comparative interpretation of treatment effects is therefore based on the adjusted between-group analyses presented in Table 3, while the within-group model-based estimates are provided in Supplementary Table 6.

**TABLE 3:** Summary of study retention, protocol adherence, and safety outcomes by group.

Outcome measure	Group A (TCM decoction)	Group B (acupoint Stim.)	Group C (combined therapy)	Group D (standard rehab.)
Enrolled	53 (100%)	53 (100%)	53 (100%)	53 (100%)
Completed 8-week follow-up	45 (84.9%)	47 (88.7%)	47 (88.7%)	47 (88.7%)
Dropouts, n (%)	8 (15.1%)	6 (11.3%)	6 (11.3%)	6 (11.3%)
*Reasons for dropout:*				
- Personal/family reasons/poor awareness	5	4	5	5
- Loss to follow-up/unreachable	2	2	1	1
- Dissatisfaction with efficacy	1	0	0	0
- Withdrawal due to adverse events	0	0	0	0
Protocol adherence and fidelity				
Mean decoction adherence rate (%)	89.7%	N/A	93.1%	N/A
Met > 80% decoction threshold, n	38	N/A	43	N/A
Formula modified by physician, n	4	N/A	3	N/A
Mean sessions completed (out of 16)	N/A	14.4	14.6	14.2
Met > 75% (≥ 12) session threshold, n	N/A	43	43	44
Dual adherence (> 80% decoction > 75% session)	N/A	N/A	40 (85.1%)	N/A
Safety profile (adverse events)				
Severe adverse events (SAEs), n	0	0	0	0
Mild diarrhea, n	1	0	1	0
Mild skin rash, n	1	1	1	0

No withdrawals due to adverse events were recorded. Breastfeeding safety was monitored throughout the study, with zero adverse signals reported related to infant health or lactation.

### 3.4 Longitudinal between-group comparisons (continuous variables)

*P*-values for the prespecified primary endpoints were adjusted for multiplicity using the Holm procedure, whereas *p*-values for secondary outcomes are presented as nominal values and should be interpreted as supportive rather than definitive.

For continuous outcomes assessed at baseline and week 8, between-group differences were evaluated using adjusted models that accounted for the non-random, patient-preference allocation. Post-treatment outcomes were compared across groups using analysis of covariance (ANCOVA), with treatment group as the main factor and adjustment for the corresponding baseline value and prespecified covariates, including age, body mass index, hemoglobin, education level, postpartum days, postpartum weight change relative to pre-pregnancy, breastfeeding status, and frequency of self-training. Where evidence of heteroscedasticity was identified, robust inference procedures were applied.

Adjusted pairwise comparisons showed that Group C had greater improvement in vaginal dynamic pressure than Group A [adjusted mean difference (MD), –3.595; 95% CI, –5.202 to –1.987; adjusted *p* < 0.00001] and Group B (MD, –2.212; 95% CI, –3.804 to –0.620; adjusted *p* = 0.033984), whereas the difference between Group C and Group D was not statistically significant (MD, 1.297; 95% CI, –0.286 to 2.881; adjusted *p* = 0.376560).

For BSD, Group C also showed greater improvement than Group A (MD, –0.172; 95% CI, –0.265 to –0.078; adjusted *p* = 0.002090) and Group B (MD, –0.246; 95% CI, –0.339 to –0.152; adjusted *p* < 0.00001). In addition, Group D showed greater improvement than Group B (MD, –0.139; 95% CI, –0.233 to –0.045; adjusted *p* = 0.020105), whereas no statistically significant difference was observed between Groups A and D or between Groups A and B. Full adjusted pairwise comparisons are presented in Table 4.

**TABLE 4:** Between-group comparisons of efficacy before and after treatment.

Outcome	Comparison	Effect measure	Adjusted effect estimate (95% CI)	Adjusted *p*-value
Vaginal dynamic pressure	A vs. B	MD	-1.383 (-2.988 to 0.222)	0.330833
	A vs. C	MD	–3.595 (–5.202 to –1.987)	< 0.00001
	A vs. D	MD	–2.297 (–3.908 to –0.687)	0.027708
	B vs. C	MD	–2.212 (–3.804 to –0.620)	0.033984
	B vs. D	MD	–0.914 (–2.511 to 0.682)	0.675713
	C vs. D	MD	1.297 (-0.286 to 2.881)	0.376560
BSD	A vs. B	MD	0.074 (–0.019 to 0.168)	0.407027
	A vs. C	MD	–0.172 (–0.265 to –0.078)	0.002090
	A vs. D	MD	–0.065 (–0.159 to 0.029)	0.529901
	B vs. C	MD	–0.246 (–0.339 to –0.152)	< 0.00001
	B vs. D	MD	–0.139 (–0.233 to –0.045)	0.020105
	C vs. D	MD	0.107 (0.014–0.200)	0.110675
ICIQ-UI-SF score	A vs. B	RR	1.011 (0.952–1.075)	0.982350
	A vs. C	RR	1.661 (1.502–1.837)	< 0.00001
	A vs. D	RR	1.361 (1.253–1.478)	< 0.00001
	B vs. C	RR	1.642 (1.489–1.812)	< 0.00001
	B vs. D	RR	1.346 (1.242–1.458)	< 0.00001
	C vs. D	RR	0.819 (0.780–0.860)	< 0.00001
POP-Q stage	A vs. B	OR	0.563 (0.559–0.568)	< 0.00001
	A vs. C	OR	0.891 (0.884–0.897)	< 0.00001
	A vs. D	OR	0.283 (0.281–0.285)	< 0.00001
	B vs. C	OR	1.581 (1.565–1.598)	< 0.00001
	B vs. D	OR	0.503 (0.498–0.508)	< 0.00001
	C vs. D	OR	0.318 (0.315–0.321)	< 0.00001
TCM syndrome score	A vs. B	MD	-1.150 (-1.647 to -0.653)	< 0.00001
	A vs. C	MD	2.314 (1.816–2.813)	< 0.00001
	A vs. D	MD	–0.973 (–1.473 to –0.473)	0.000904
	B vs. C	MD	3.465 (2.971–3.959)	< 0.00001
	B vs. D	MD	0.177 (–0.318 to 0.673)	0.896482
	C vs. D	MD	–3.287 (–3.777 to –2.797)	< 0.00001

MD, mean difference; RR, response ratio; OR, odds ratio; BSD, bladder neck–symphysis distance; ICIQ-UI-SF, International Consultation on Incontinence Questionnaire-Urinary Incontinence Short Form. For continuous outcomes, values are adjusted mean differences with 95% confidence intervals. For ICIQ-UI-SF, values are model-based response ratios with 95% confidence intervals. For POP-Q stage, values are adjusted odds ratios from ordinal regression models. Adjusted *p*-values account for multiple pairwise comparisons.

#### 3.4.1 Outcomes meeting homogeneity of variance

For continuous outcomes that satisfied the homogeneity of variance assumption, between-group differences at week 8 were evaluated using adjusted analysis of covariance (ANCOVA) models. Post-treatment outcomes were compared across groups with treatment group as the main factor, while adjusting for the corresponding baseline value and prespecified covariates to account for the non-random, patient-preference allocation and potential baseline imbalance. Adjusted pairwise between-group comparisons were performed using Tukey correction for multiple comparisons. Effect estimates are reported as adjusted mean differences with 95% confidence intervals, and the full adjusted pairwise comparisons are presented in Table 4.

##### 3.4.1.1 Between-group comparison of vaginal dynamic pressure (baseline vs. post-treatment)

**FIGURE 3:**
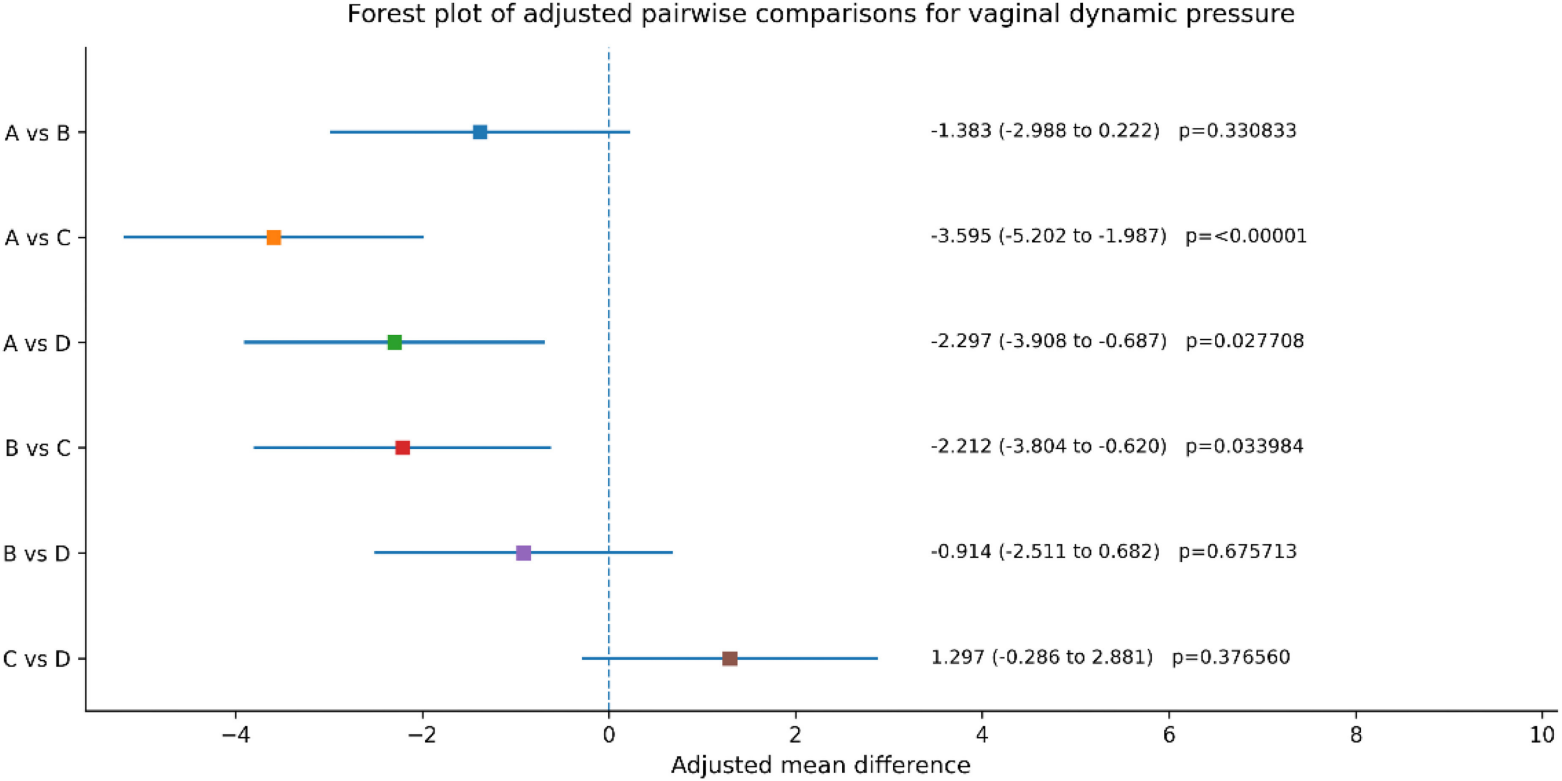
Between group comparison of vaginal dynamic pressure before and post treatment.

After covariate adjustment and multiplicity control, all groups showed improvement in vaginal dynamic pressure over the 8-week period. Adjusted pairwise comparisons demonstrated that Group C had greater improvement than Group A [adjusted mean difference (MD), 3.595; 95% CI, 1.987–5.202; adjusted *p* < 0.00001) and Group B (MD, 2.212; 95% CI, 0.620–3.804; adjusted *p* = 0.033984). Group D also showed greater improvement than Group A (MD, 2.297; 95% CI, 0.687–3.908; adjusted *p* = 0.027708). No statistically significant differences were observed for A versus B, B versus D, or C versus D after Tukey adjustment. Overall, these findings suggest that the combination therapy was associated with greater improvement in vaginal dynamic pressure than Groups A and B, whereas the difference between the combination therapy and standard electrical stimulation/biofeedback was not statistically significant (Figure 3 and Table 4).

##### 3.4.1.2 Between group comparison of BSD before and post treatment

**FIGURE 4:**
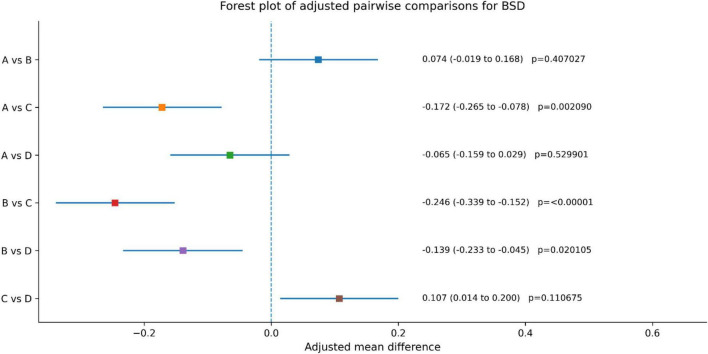
Between group comparison of BSD before and post treatment.

After covariate adjustment and Tukey correction for multiple comparisons, BSD improved in all groups over the 8-week period. Adjusted pairwise comparisons showed that Group C had greater improvement than Group A [adjusted mean difference (MD), 0.172; 95% CI, 0.078–0.265; adjusted *p* = 0.002090) and Group B (MD, 0.246; 95% CI, 0.152–0.339; adjusted *p* < 0.00001). Group D also showed greater improvement than Group B (MD, 0.139; 95% CI, 0.045–0.233; adjusted *p* = 0.020105). No statistically significant differences were observed for A versus B, A versus D, or C versus D after Tukey adjustment. Overall, these findings indicate that the combination therapy was associated with greater improvement in BSD than Groups A and B, whereas the difference between the combination therapy and Group D was not statistically significant (Figure 4 and Table 4).

#### 3.4.2 Between-group comparisons for variables that violated the homogeneity of variance assumption

For outcomes showing evidence of heteroscedasticity, between-group comparisons were performed using adjusted regression models with heteroscedasticity-robust inference. To account for the non-random, patient-preference allocation, post-treatment outcomes were compared across groups with adjustment for the corresponding baseline value and prespecified covariates. Outcomes analyzed under this framework included the urinary incontinence questionnaire score (ICIQ-UI-SF) and the TCM syndrome score. Effect estimates are reported with 95% confidence intervals, and the adjusted pairwise comparisons are presented in Table 4.

##### 3.4.2.1 Urinary incontinence questionnaire score (ICIQ-UI-SF)

**FIGURE 5:**
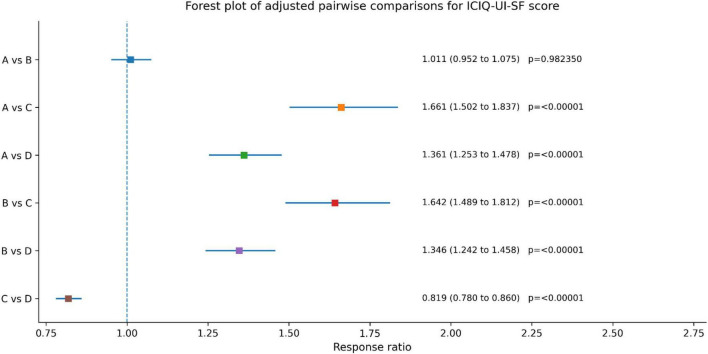
Between group comparison of Urinary incontinence questionnaire score before and post treatment.

Between-group differences in urinary incontinence symptoms were summarized using response ratios (RRs), with values greater than 1 indicating greater improvement in the first-listed group and values less than 1 indicating greater improvement in the second-listed group. Adjusted pairwise comparisons showed that Group C had greater improvement than Group A (RR, 1.661; 95% CI, 1.502–1.837; adjusted *p* < 0.00001) and Group B (RR, 1.642; 95% CI, 1.489–1.812; adjusted *p* < 0.00001). Group D also showed greater improvement than Group A (RR, 1.361; 95% CI, 1.253–1.478; adjusted *p* < 0.00001) and Group B (RR, 1.346; 95% CI, 1.242–1.458; adjusted *p* < 0.00001). No statistically significant difference was observed between Groups A and B (RR, 1.011; 95% CI, 0.952–1.075; adjusted *p* = 0.982350). Compared with Group D, Group C also showed greater improvement (equivalently, RR for C vs. D, 0.819; 95% CI, 0.780–0.860; adjusted *p* < 0.00001), consistent with a more favorable reduction in ICIQ-UI-SF score in Group C (Figure 5 and Table 4).

##### 3.4.2.2 TCM syndrome scoring

**FIGURE 6:**
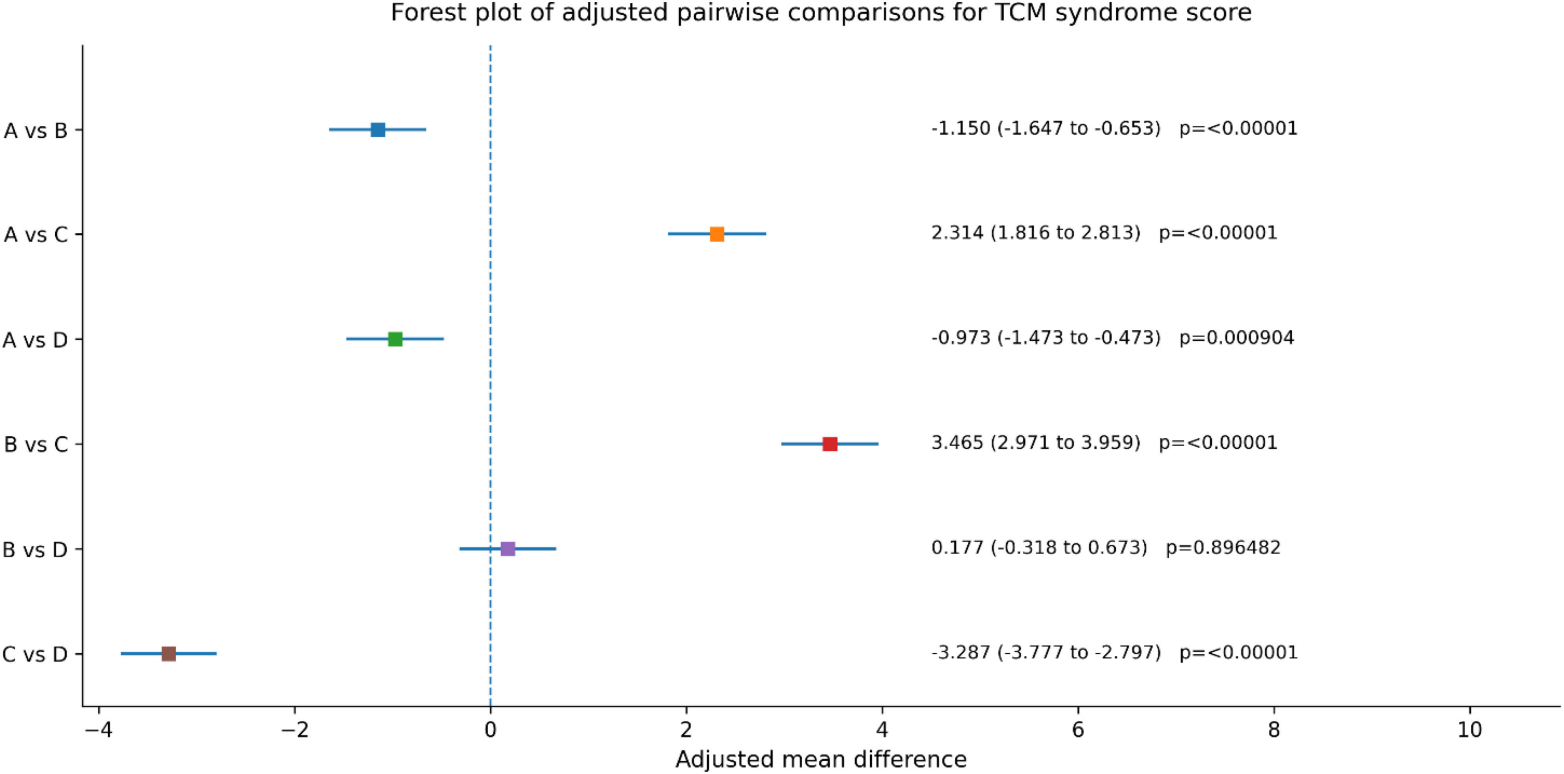
Between group comparison of TCM syndrome scoring before and after treatment.

Adjusted pairwise comparisons with heteroscedasticity-robust inference showed significant between-group differences in TCM syndrome score reduction. Group C demonstrated greater improvement than Group A [adjusted mean difference (MD), 2.314; 95% CI, 1.816–2.813; adjusted *p* < 0.00001), Group B (MD, 3.465; 95% CI, 2.971–3.959; adjusted *p* < 0.00001), and Group D (MD, 3.287; 95% CI, 2.797–3.777; adjusted *p* < 0.00001). Group A also showed greater improvement than Group D (MD, 0.973; 95% CI, 0.473–1.473; adjusted *p* = 0.000904), whereas no statistically significant difference was observed between Groups B and D (MD, 0.177; 95% CI, -0.318 to 0.673; adjusted *p* = 0.896482). Overall, all groups showed improvement in TCM syndrome score, with the greatest reduction observed in Group C (Figure 6 and Table 4).

### 3.5 Between group comparison of ordinal outcome—POP-Q staging

Statistical method: POP-Q stage was analyzed as an ordered categorical outcome using a proportional odds model (ordinal logistic regression). Because treatment allocation was based on patient preference rather than randomization, treatment group was entered as the main predictor, with adjustment for prespecified covariates, including age, body mass index, educational level, calcium supplementation, baseline vaginal dynamic pressure, and baseline BSD. The proportional odds assumption was evaluated using Brant-type tests and diagnostic procedures. Pairwise group contrasts were derived from the fitted model and are reported as adjusted odds ratios (ORs) with 95% confidence intervals; multiplicity for the primary ordinal endpoint was controlled using Holm-adjusted *p*-values.

**FIGURE 7:**
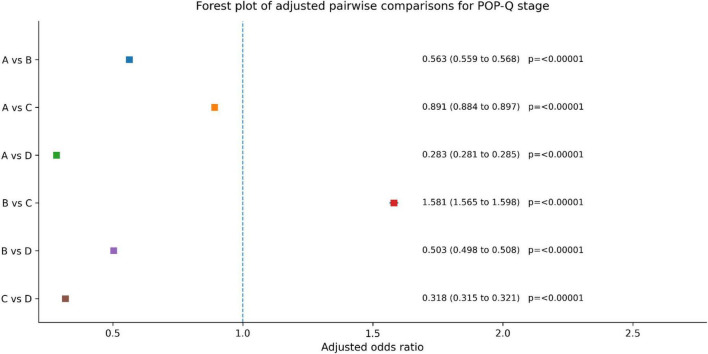
POP-Q stage improvement status.

Results: After adjustment, POP-Q stage differed significantly between groups. Pairwise comparisons showed significant differences between Group C and Group A (OR, 0.891; 95% CI, 0.884–0.897; adjusted *p* < 0.00001), Group B and Group C (OR, 1.581; 95% CI, 1.565–1.598; adjusted *p* < 0.00001), and Group C and Group D (OR, 0.318; 95% CI, 0.315–0.321; adjusted *p* < 0.00001). Additional significant differences were also observed between Group A and Group B (OR, 0.563; 95% CI, 0.559–0.568; adjusted *p* < 0.00001), Group A and Group D (OR, 0.283; 95% CI, 0.281–0.285; adjusted *p* < 0.00001), and Group B and Group D (OR, 0.503; 95% CI, 0.498–0.508; adjusted *p* < 0.00001). Because lower POP-Q stage indicates clinical improvement, these findings support significant between-group differences in post-treatment POP-Q distribution, with Group C showing the most favorable overall outcome pattern among the four groups (Figure 7 and Table 4).

#### 3.5.1 Interpretation summary

POP-Q staging, analyzed with a proportional odds (ordinal logistic) model, showed significant differences between all treatment groups. The combination therapy (Group C) achieved the greatest improvement in pelvic organ support, followed by the TCM decoction group (A), the acupoint stimulation group (B), and finally electrical stimulation/biofeedback (D). These findings consistently support Group C as the most favorable post-treatment POP-Q pattern.

### 3.6 Sensitive analysis

To examine the robustness of the main findings, prespecified sensitivity analyses were performed using alternative adjustment strategies, including propensity score–based weighted analyses and multiple imputation for missing data. For the ordinal primary outcome, POP-Q stage, adjusted post-treatment pairwise comparisons yielded results consistent with the primary proportional odds model, supporting the stability of the observed between-group differences and the most favorable post-treatment pattern in Group C.

For the remaining outcomes, robustness was assessed through the predefined sensitivity framework rather than through additional *ad hoc post-hoc* tests. Specifically, continuous outcomes were re-evaluated using the same covariate structure across complete-case and multiply imputed datasets, and the direction and magnitude of the treatment effects were generally consistent. In addition, propensity score–based weighted analyses produced broadly similar patterns of between-group differences, supporting the robustness of the primary findings.

### 3.7 Statistical power analysis, power estimates assume α = 0.05 and Cohen’s f effect sizes

**FIGURE 8:**
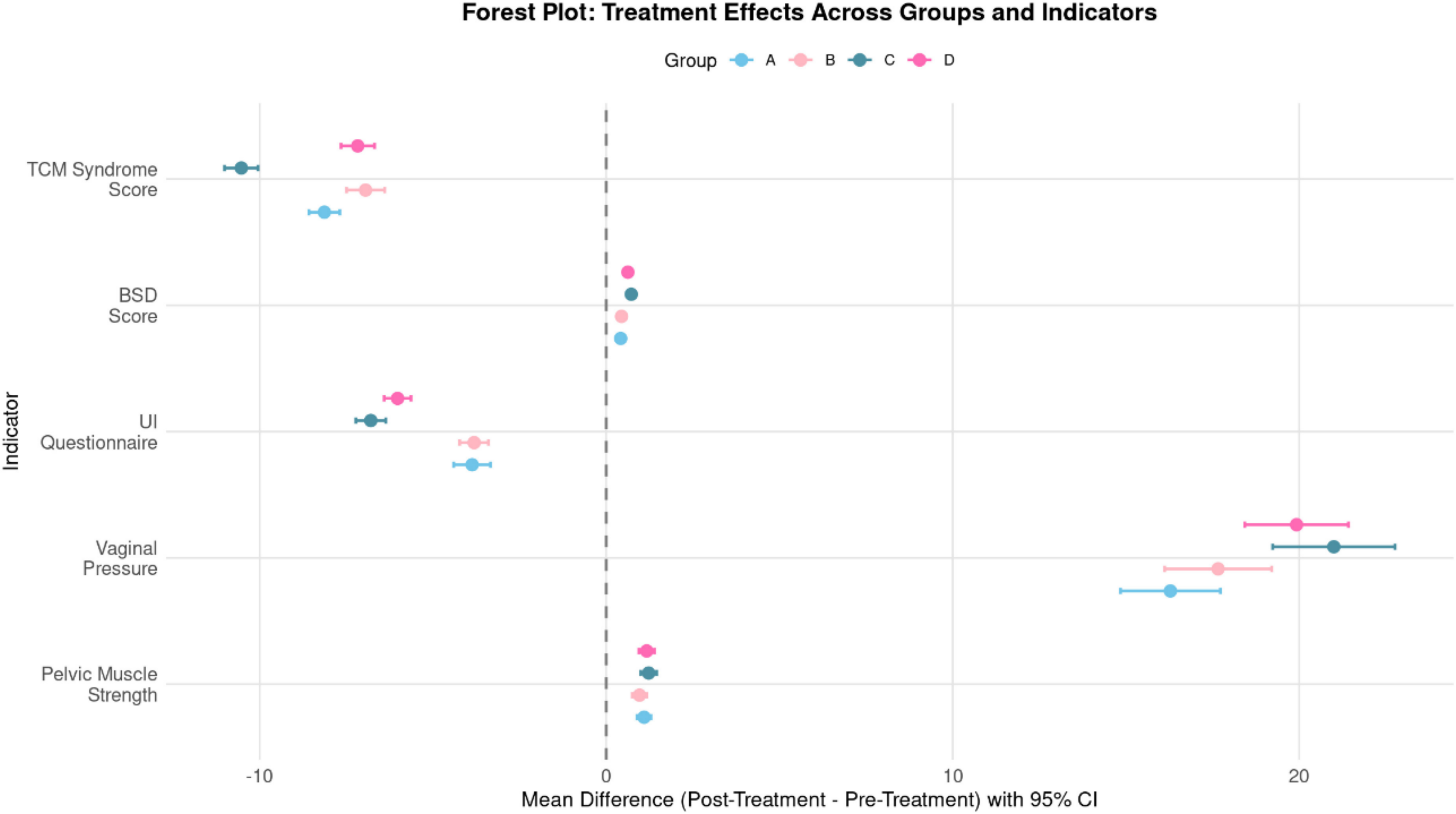
Forest plot of treatment effects across groups and indicators.

As described in the Methods, the planned sample size was based on a four-group comparison under the assumption of a moderate overall effect size (Cohen’s *f* = 0.25), a two-sided α of 0.05, and 80% power, yielding a target total sample size of 212 participants. On this basis, the achieved sample size was considered adequate for detecting moderate between-group differences under the original design assumptions. However, given the non-randomized, patient-preference allocation and the use of adjusted regression models, interpretation should rely primarily on the estimated effect sizes, confidence intervals, and sensitivity analyses rather than on *post hoc* power considerations (Figure 8).

### 3.8 Supplementary results weight diagnostics balance and robustness of effect estimates

Weight diagnostics indicated acceptable stability after trimming. Stabilized inverse probability of treatment weights had a median of 0.835 with the first and ninety ninth percentiles 0.412 and 2.611 and a maximum of 4.243. After trimming at the first and ninety ninth percentiles, weights were bounded within 0.412–2.611. Generalized overlap weights were more tightly bounded with a median of 0.726 and a range of 0.161–2.260. These summaries are reported in Supplementary Table 1.

Baseline imbalance was substantial prior to weighting. Fourteen of fifteen prespecified covariates had a maximum absolute standardized mean difference above 0.10 in the unweighted sample. Weighting improved balance, reducing covariates with maximum absolute standardized mean difference above 0.10 to nine of fifteen under both trimmed inverse probability weighting and overlap weighting. Balance details and the overall balance summary are shown in Supplementary Tables 2, 3.

Across outcomes, sensitivity analyses yielded effect estimates consistent in direction and magnitude with the primary covariate adjusted models. For the urinary incontinence questionnaire score, the Group C versus Group D difference remained robust across estimators, with an adjusted mean difference of minus 0.995 with a 95 percent confidence interval from minus 1.387 to minus 0.603 in the primary adjusted model, minus 1.083 from minus 1.517 to minus 0.648 in trimmed inverse probability weighting doubly robust models, and minus 1.056 from minus 1.480 to minus 0.633 under overlap weighted doubly robust models. For the TCM syndrome score, Group C versus Group D differences were similarly stable, with an adjusted mean difference of minus 3.321 from minus 4.024 to minus 2.618 in the primary model and minus 3.583 from minus 4.405 to minus 2.761 in overlap weighted doubly robust models. In contrast, the Group C versus Group D comparison for vaginal dynamic pressure was not statistically significant under any approach. BSD score differences for Group C versus Group D were small but remained statistically significant across weighting approaches. Full results for continuous outcomes are provided in Supplementary Table 4.

Weighting improved baseline balance and this is visualized in Supplementary Figure 1. Across outcomes, sensitivity analyses yielded effect estimates consistent with the primary covariate adjusted models, and the cross method consistency is summarized in Supplementary Figure 2.

### 3.9 Retention, treatment adherence, and safety

Throughout the 8-week intervention period, treatment fidelity and safety were systematically monitored. Of the 212 enrolled participants, 186 completed the study, yielding an overall retention rate of 87.7%. The retention rates were comparable across groups: 84.9% (45/53) in Group A, and 88.7% (47/53) in Groups B, C, and D, respectively. A total of 26 participants dropped out, with the vast majority of dropouts attributed to non-medical reasons such as loss to follow-up, personal/family reasons, or unreachability (*n* = 25). Only one participant in Group A withdrew due to dissatisfaction with the treatment efficacy. Crucially, no participants withdrew due to adverse events.

Among the retained participants, protocol adherence was excellent. In Group A, the mean adherence rate to the herbal decoction was 89.7%, with 38 out of 45 participants meeting the > 80% protocol threshold. Minor, physician-guided modifications to the formula were made for 4 patients in Group A and 3 patients in Group C. For the physical interventions, participants in Group B completed a mean of 14.4 out of 16 scheduled acupoint sessions, while those in Group D completed a mean of 14.2 standard rehabilitation sessions. In the combined therapy group (Group C), the mean decoction adherence was 93.1%, and participants completed an average of 14.6 acupoint sessions, with 85.1% (40/47) meeting the dual adherence thresholds for both treatments.

Regarding safety, no severe adverse events (SAEs) occurred during the study. Mild, self-limiting adverse events were rare and included mild skin rashes (*n* = 3; 1 each in Groups A, B, and C) and mild diarrhea (*n* = 2, 1 each in Groups A and C). None of these mild side effects required major medical intervention or treatment discontinuation. Crucially, safety monitoring specifically included breastfeeding-related observations; no adverse events or negative signals concerning breastfeeding performance or neonatal health were identified across all four groups. A detailed summary of retention, protocol adherence, and safety profiles by group is provided in Table 3.

**FIGURE 9:**
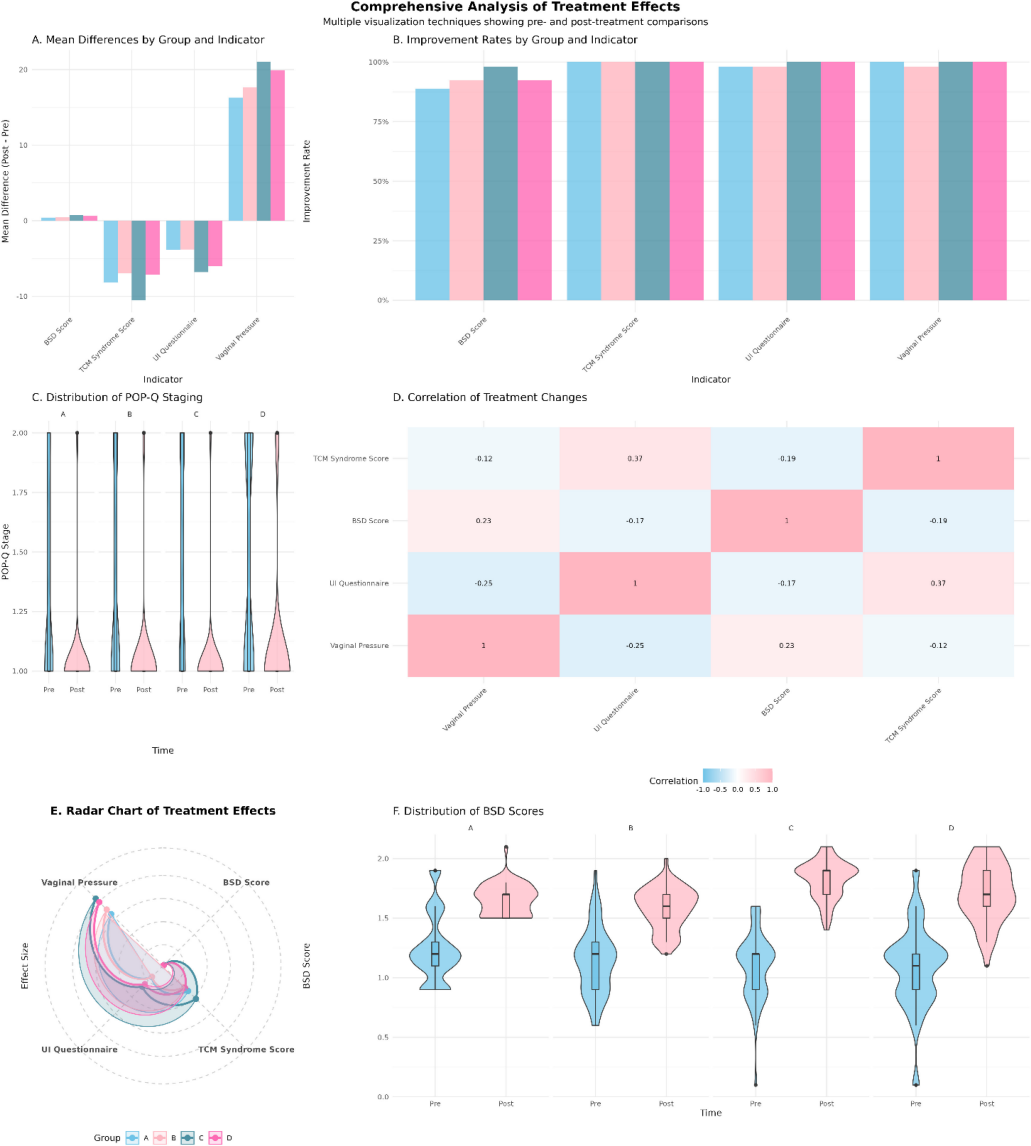
Composite overview of treatment effects: multi-indicator, between-group comparisons.

In summary (Figure 9): All groups improved from baseline. Functional vaginal pressure increased, symptom scales (urinary incontinence questionnaire score (UI questionnaire) and TCM syndrome score) decreased, POP-Q distributions shifted slightly toward lower stages, and BSD showed only modest short-term change. Correlations were coherent—vaginal pressure gains were inversely related to symptom scores and only weakly related to BSD. Overall effect magnitude follows Combination therapy (Group C) ≥ Rehabilitation (Group D) > Acupoint (B) ≈ TCM (A), confirming Group C as most effective with Group D close behind.

For a detailed representation of the absolute post-treatment distribution and mean values with 95% CIs for each group, please refer to Supplementary Figure 4.

## 4 Discussion

Focusing on postpartum pelvic floor dysfunction and improving pelvic floor function is one of the key objectives of postpartum rehabilitation. Research indicates that pregnancy and childbirth can exert pressure, stretching, or tearing on nerves, muscles, and connective tissues, leading to pelvic floor damage (6). The current standard treatment involves electrical stimulation biofeedback therapy, which requires patients to visit the hospital for treatment using specific transvaginal devices (20). However, postpartum women often find it inconvenient to leave home and may experience discomfort due to traditional beliefs and unregulated postpartum hormonal levels, such as vaginal congestion, which can cause pain during treatment. This discomfort may lead to fear, affect compliance, and ultimately reduce the effectiveness of treatment.

TCM emphasizes the coordination of organ functions and the integration of internal and external therapies in postpartum recovery, The results indicate that, in addition to common symptoms of pelvic floor dysfunction, patients also exhibited significant signs of “Qi descent” according to TCM. In TCM, postpartum disorders caused by childbirth are known as “postpartum illness.” The “Golden Mirror of Medicine: Essential Gynecology” mention (21) “Pelvic prolapse in women can result from damage to the uterine network, excessive exertion during delivery, or Qi deficiency leading to descent. akin to the ancient condition known as hernia.” Postpartum Qi deficiency may impair the supportive structure of the uterus, potentially leading to uterine prolapse. Insufficient kidney Qi can result in bladder dysfunction, causing postpartum urinary incontinence, while kidney Yang deficiency might weaken bladder Qi transformation, leading to frequent urination. These issues are closely related to the weakened state of Qi and blood in postpartum women. Literature reports that among women who deliver vaginally, 37.5% exhibit a Qi deficiency constitution, which is significantly higher than other types (22). Regarding the potential pharmacological mechanisms, modern studies suggest that the key components of the Dabu-Yuanjian Tisheng formula, such as Radix Rehmanniae and Radix Astragali, may facilitate pelvic floor recovery by promoting collagen synthesis, modulating fibroblast activity, and enhancing the contractile properties of pelvic floor muscle fibers. These biological actions, combined with the immunomodulatory effects of the formula, potentially strengthen the supportive connective tissues of the pelvic floor, providing a physiological basis for the observed clinical improvements. Our study also found that in patients with postpartum pelvic floor dysfunction, the primary TCM pathomechanism is spleen deficiency leading to insufficient central Qi, resulting in Qi sinking, instability of the Chong and Ren meridians, and imbalance of the Dai meridian, ultimately causing organ prolapse.

Acupuncture is a traditional external therapy within Chinese medicine. In clinical practice, it has been observed that postpartum patients often exhibit fear toward direct needling, which can hinder the progress of treatment. This study modifies the traditional acupuncture approach by employing suction-based acupoint stimulation with low-frequency therapy to reduce patient anxiety and enhance compliance. The selection of acupoints was guided by the principles of near and distant treatment, including the adjacent uterine-related points such as the “Uterus” (似穴) and “Guanyuan” (关元), as well as the “Sanyinjiao” (三阴交) point on the Foot Taiyin Spleen meridian, and the “Shen Shu” (肾俞) point on the Foot Taiyang Bladder meridian. (23)

The “Uterus” point is an extra-regular acupoint suitable for treating organ prolapse. “Guanyuan,” belonging to the Ren Meridian, has the functions of tonifying Yuan Qi and consolidating the essence, which makes it particularly effective for persons with deficiency in vital energy. “Sanyinjiao” is frequently used for regulating the female reproductive system and improving endocrine function. “Shen Shu” is associated with the Bladder meridian and the Governor Vessel, aiding in the regulation of bladder Qi and Yuan Qi. According to research (24), acupuncture at points on the Bladder meridian, the “Shang Yang” (上阳) and “Zhong Liao” (中髎) points, also has been recommended for treating stress urinary incontinence in women with Kidney Qi deficiency, as these points help regulate bladder meridian Qi, warm and invigorate the Yang of the Governor Vessel, thereby strengthening retention functions. The integration of modern medicine with TCM provides new approaches and methods for clinical treatment. TCM advocates for early intervention, addressing issues at their onset. Timely rehabilitation following pregnancy and childbirth-related damage can promote rapid neuromuscular repair and prevent long-term complications.

This study focuses on postpartum women with pelvic floor dysfunction, guided by the holistic principles of TCM. It explores an integrated approach combining traditional Chinese herbal medicine with surface acupoint stimulation. The evaluation encompasses multiple aspects, including pelvic anatomical structure, pelvic floor muscle strength, vaginal dynamic pressure, symptoms of urinary incontinence, and TCM symptom manifestations. Through the POP-Q scoring system in this study, we assessed vaginal dynamic pressure, and pelvic organ positioning from multiple angles. We also conducted a comparative analysis of scores from the International Consultation on ICIQ-SF and the quantitative scoring of TCM syndromes. For postpartum pelvic floor rehabilitation in this dataset, Group C was associated with more favorable and more consistent improvement patterns; D is a useful comparator but generally does not match C; A and B alone show limited incremental benefit versus each other after multiplicity adjustment. Changes before and after treatment were systematically compared. The results suggest that the selection of the combined Dabu-Yuanjian Tisheng formula and acupoint surface stimulation is associated with enhanced pelvic floor muscle strength, increased vaginal dynamic pressure, improvements in pelvic organ prolapse staging, reduced incidence of urinary incontinence, and alleviation of postpartum Qi deficiency, thereby promoting overall postpartum recovery. This presents a novel perspective for the integrated application of traditional Chinese medicine and Western medicine in the treatment of postpartum pelvic floor dysfunction. Clinical relevance should be interpreted alongside statistical significance. For outcomes such as vaginal dynamic pressure and BSD, although several between-group differences reached statistical significance, well-established minimal clinically important differences are not currently available in postpartum pelvic floor dysfunction. We therefore interpreted these findings on the basis of the magnitude and direction of the observed effects, the corresponding 95% confidence intervals, and the consistency of the results across related clinical endpoints. Accordingly, these findings should be viewed as supportive evidence of comparative benefit rather than as stand-alone proof of definitive clinical superiority.

Limitations: Several limitations should be acknowledged. First, although we implemented measures to reduce assessment bias, including separation of outcome assessors from the treatment team and standardized measurement procedures, the open-label design may still have introduced performance and measurement bias, particularly for subjective outcomes. In addition, formal intra-rater and inter-rater reliability statistics for examiner-dependent outcomes, such as the Modified Oxford Scale, were not prospectively collected. Second, no placebo or pure no-treatment control group was included. Because postpartum pelvic floor function has a natural recovery trajectory, the within-group improvements observed across all four arms likely reflect a combination of active intervention effects, spontaneous physiological recovery, and unmeasured co-interventions such as informal home pelvic floor exercises. Third, the non-randomized, patient-preference allocation introduced substantial baseline imbalance, selection bias, and confounding by indication, all of which limit causal inference. Participants who selected the TCM-based or combined interventions may also have held stronger prior beliefs regarding their effectiveness, which could have influenced adherence, self-care behaviors, and subjective patient-reported outcomes such as the ICIQ-UI-SF and TCM syndrome score. Although multivariable adjustment and propensity score-based sensitivity analyses were used to reduce bias from measured confounders, residual confounding due to unmeasured behavioral, cultural, and psychological factors cannot be excluded. The non-randomized, patient-preference allocation introduced substantial baseline imbalance, selection bias, and confounding by indication, all of which limit causal inference (Supplementary Figure 3). Fourth, although the TCM syndrome score was retained as a supportive secondary outcome to capture syndrome-related symptom changes within the integrative medicine framework, its international interpretability remains limited, and it has not been broadly validated specifically for postpartum pelvic floor dysfunction. It should therefore be interpreted alongside, rather than in place of, the objective and internationally recognized clinical endpoints. Finally, this was a single-center study conducted within a specific cultural and healthcare context in which acceptance of traditional Chinese medicine may have been relatively high, potentially limiting the generalizability of the findings to other settings and populations.

Future studies should incorporate multidisciplinary collaboration and adopt designs that better distinguish treatment effects from natural recovery, such as pragmatic trials, preference-based randomized designs, or longer-term follow-up with repeated assessments. Future studies should incorporate multidisciplinary collaboration (e.g., gynecology, rehabilitation medicine, pelvic floor physiotherapy, and traditional Chinese medicine) and adopt designs that better distinguish treatment effects from natural recovery, such as pragmatic trials, preference-based randomized designs, or longer-term follow-up with repeated assessments.

## Data Availability

The datasets presented in this study can be found in online repositories. The names of the repository/repositories and accession number(s) can be found at: 10.5281/zenodo.18169662.

## Footnotes

1
https://posit.cloud/content/11057733
