# Supplementary material for: Clinical effects of integrated Chinese medicine therapy for postpartum pelvic floor dysfunction: a prospective patient-preference cohort study
Source: Front Med (Lausanne). 2026 Apr 2;13:1782551. doi: 10.3389/fmed.2026.1782551 (PMC13083059; doi:10.3389/fmed.2026.1782551)
Supplement: Supplementary file 1 [file Data_Sheet_1.docx]

**Supplemental instrument**

Table S1 weight summary

| scheme | min | p01 | p50 | p99 | max | mean |
| --- | --- | --- | --- | --- | --- | --- |
| IPTW (stabilized) | 0.361503 | 0.412249 | 0.835461 | 2.610954 | 4.242933 | 0.990627 |
| IPTW (trim 1/99) | 0.412249 | 0.413706 | 0.835461 | 2.606759 | 2.610954 | 0.977116 |
| Overlap weights | 0.161308 | 0.202088 | 0.725723 | 1.965728 | 2.259656 | 0.796037 |

Table S2 Balance MaxPairwiseSMD

| Covariate | Max\|SMD\| Unweighted | Max\|SMD\| IPTW(trim) | Max\|SMD\| Overlap |
| --- | --- | --- | --- |
| Age | 0.174738 | 0.071104 | 0.042547 |
| Degree.of.education | 0.339887 | 0.130657 | 0.119413 |
| BMI | 0.488614 | 0.010291 | 0.069642 |
| Postpartum.days | 0.136307 | 0.056669 | 0.035252 |
| Number.of.pregnancies | 0.06663 | 0.183938 | 0.189972 |
| Haemoglobin | 0.229651 | 0.152631 | 0.088999 |
| Taking.calcium.tablets | 0.349563 | 0.052747 | 0.087581 |
| Weight.change.postpartum.compared.to.pre.pregnancy | 0.414045 | 0.13841 | 0.169031 |
| Lactation | 0.168133 | 0.074088 | 0.113032 |
| Pelvic.floor.muscle.strength.type.pre.treatment | 0.147506 | 0.170512 | 0.127442 |
| Vaginal.dynamic.pressure.pre.treatment | 0.363315 | 0.185886 | 0.205748 |
| Urinary.incontinence.questionnaire.score.pre.treatment | 0.275698 | 0.132159 | 0.126349 |
| BSD.pre.treatment | 0.506422 | 0.198432 | 0.149119 |
| TCM.Syndrome.Scoring.pre.treatment | 0.183956 | 0.167466 | 0.182901 |
| POP.Q.stag1ng.pre.treatment | 0.362033 | 0.047038 | 0.077117 |

Table S3 Balance Summary

| scheme | n(Max\|SMD\|>0.10) |
| --- | --- |
| Unweighted | 14 |
| IPTW (trim 1/99) | 9 |
| Overlap weights | 9 |

Table S4. Sensitivity analyses for continuous outcomes (Groups A–C vs Group D).

| Outcome | Direction | Contrast | Method | Effect (95% CI) | P |
| --- | --- | --- | --- | --- | --- |
| Vaginal dynamic pressure | MD (higher=better) | A vs D | ANCOVA (unweighted, adjusted) | -2.111 (-3.848, -0.374) | 0.017228 |
| Vaginal dynamic pressure | MD (higher=better) | B vs D | ANCOVA (unweighted, adjusted) | -1.000 (-2.683, 0.683) | 0.244196 |
| Vaginal dynamic pressure | MD (higher=better) | C vs D | ANCOVA (unweighted, adjusted) | 1.313 (-0.448, 3.074) | 0.143806 |
| Vaginal dynamic pressure | MD (higher=better) | A vs D | IPTW(trim), weighted-only | -2.109 (-3.915, -0.303) | 0.022087 |
| Vaginal dynamic pressure | MD (higher=better) | B vs D | IPTW(trim), weighted-only | -0.741 (-2.359, 0.878) | 0.369554 |
| Vaginal dynamic pressure | MD (higher=better) | C vs D | IPTW(trim), weighted-only | 1.155 (-0.440, 2.749) | 0.155752 |
| Vaginal dynamic pressure | MD (higher=better) | A vs D | IPTW(trim), doubly robust | -2.159 (-4.038, -0.280) | 0.024306 |
| Vaginal dynamic pressure | MD (higher=better) | B vs D | IPTW(trim), doubly robust | -0.879 (-2.579, 0.821) | 0.310977 |
| Vaginal dynamic pressure | MD (higher=better) | C vs D | IPTW(trim), doubly robust | 1.093 (-0.577, 2.763) | 0.199696 |
| Vaginal dynamic pressure | MD (higher=better) | A vs D | Overlap weights, weighted-only | -2.144 (-3.805, -0.483) | 0.011393 |
| Vaginal dynamic pressure | MD (higher=better) | B vs D | Overlap weights, weighted-only | -0.951 (-2.531, 0.629) | 0.238288 |
| Vaginal dynamic pressure | MD (higher=better) | C vs D | Overlap weights, weighted-only | 0.871 (-0.646, 2.389) | 0.260475 |
| Vaginal dynamic pressure | MD (higher=better) | A vs D | Overlap weights, doubly robust | -2.217 (-4.015, -0.419) | 0.015655 |
| Vaginal dynamic pressure | MD (higher=better) | B vs D | Overlap weights, doubly robust | -1.010 (-2.705, 0.685) | 0.242745 |
| Vaginal dynamic pressure | MD (higher=better) | C vs D | Overlap weights, doubly robust | 0.820 (-0.809, 2.450) | 0.323896 |
| Urinary incontinence score | MD (lower=better) | A vs D | ANCOVA (unweighted, adjusted) | 1.967 (1.457, 2.476) | 3.83E-14 |
| Urinary incontinence score | MD (lower=better) | B vs D | ANCOVA (unweighted, adjusted) | 1.884 (1.499, 2.269) | 8.39E-22 |
| Urinary incontinence score | MD (lower=better) | C vs D | ANCOVA (unweighted, adjusted) | -0.995 (-1.387, -0.603) | 6.40E-07 |
| Urinary incontinence score | MD (lower=better) | A vs D | IPTW(trim), weighted-only | 1.733 (1.073, 2.394) | 2.71E-07 |
| Urinary incontinence score | MD (lower=better) | B vs D | IPTW(trim), weighted-only | 1.818 (1.396, 2.240) | 3.27E-17 |
| Urinary incontinence score | MD (lower=better) | C vs D | IPTW(trim), weighted-only | -1.093 (-1.526, -0.660) | 7.63E-07 |
| Urinary incontinence score | MD (lower=better) | A vs D | IPTW(trim), doubly robust | 1.762 (1.084, 2.439) | 3.44E-07 |
| Urinary incontinence score | MD (lower=better) | B vs D | IPTW(trim), doubly robust | 1.829 (1.395, 2.264) | 1.65E-16 |
| Urinary incontinence score | MD (lower=better) | C vs D | IPTW(trim), doubly robust | -1.083 (-1.517, -0.648) | 1.04E-06 |
| Urinary incontinence score | MD (lower=better) | A vs D | Overlap weights, weighted-only | 1.823 (1.245, 2.401) | 6.41E-10 |
| Urinary incontinence score | MD (lower=better) | B vs D | Overlap weights, weighted-only | 1.876 (1.456, 2.296) | 2.14E-18 |
| Urinary incontinence score | MD (lower=better) | C vs D | Overlap weights, weighted-only | -1.060 (-1.483, -0.638) | 8.81E-07 |
| Urinary incontinence score | MD (lower=better) | A vs D | Overlap weights, doubly robust | 1.847 (1.251, 2.442) | 1.20E-09 |
| Urinary incontinence score | MD (lower=better) | B vs D | Overlap weights, doubly robust | 1.887 (1.465, 2.309) | 1.92E-18 |
| Urinary incontinence score | MD (lower=better) | C vs D | Overlap weights, doubly robust | -1.056 (-1.480, -0.633) | 9.88E-07 |
| BSD score | MD (lower=better) | A vs D | ANCOVA (unweighted, adjusted) | -0.059 (-0.138, 0.020) | 0.145968 |
| BSD score | MD (lower=better) | B vs D | ANCOVA (unweighted, adjusted) | -0.140 (-0.228, -0.053) | 0.001724 |
| BSD score | MD (lower=better) | C vs D | ANCOVA (unweighted, adjusted) | 0.111 (0.029, 0.194) | 0.00835 |
| BSD score | MD (lower=better) | A vs D | IPTW(trim), weighted-only | -0.087 (-0.165, -0.008) | 0.030968 |
| BSD score | MD (lower=better) | B vs D | IPTW(trim), weighted-only | -0.178 (-0.264, -0.093) | 4.70E-05 |
| BSD score | MD (lower=better) | C vs D | IPTW(trim), weighted-only | 0.084 (0.001, 0.167) | 0.047476 |
| BSD score | MD (lower=better) | A vs D | IPTW(trim), doubly robust | -0.084 (-0.164, -0.004) | 0.039871 |
| BSD score | MD (lower=better) | B vs D | IPTW(trim), doubly robust | -0.176 (-0.264, -0.087) | 0.000102 |
| BSD score | MD (lower=better) | C vs D | IPTW(trim), doubly robust | 0.087 (0.001, 0.173) | 0.047466 |
| BSD score | MD (lower=better) | A vs D | Overlap weights, weighted-only | -0.079 (-0.157, -0.001) | 0.04804 |
| BSD score | MD (lower=better) | B vs D | Overlap weights, weighted-only | -0.174 (-0.261, -0.087) | 9.38E-05 |
| BSD score | MD (lower=better) | C vs D | Overlap weights, weighted-only | 0.091 (0.007, 0.176) | 0.034775 |
| BSD score | MD (lower=better) | A vs D | Overlap weights, doubly robust | -0.077 (-0.157, 0.003) | 0.058295 |
| BSD score | MD (lower=better) | B vs D | Overlap weights, doubly robust | -0.169 (-0.258, -0.080) | 0.000201 |
| BSD score | MD (lower=better) | C vs D | Overlap weights, doubly robust | 0.094 (0.006, 0.182) | 0.036862 |
| TCM syndrome score | MD (lower=better) | A vs D | ANCOVA (unweighted, adjusted) | -0.958 (-1.657, -0.259) | 0.00722 |
| TCM syndrome score | MD (lower=better) | B vs D | ANCOVA (unweighted, adjusted) | 0.158 (-0.556, 0.871) | 0.664926 |
| TCM syndrome score | MD (lower=better) | C vs D | ANCOVA (unweighted, adjusted) | -3.321 (-4.024, -2.618) | 2.10E-20 |
| TCM syndrome score | MD (lower=better) | A vs D | IPTW(trim), weighted-only | -1.149 (-1.922, -0.376) | 0.00358 |
| TCM syndrome score | MD (lower=better) | B vs D | IPTW(trim), weighted-only | -0.065 (-0.854, 0.723) | 0.870753 |
| TCM syndrome score | MD (lower=better) | C vs D | IPTW(trim), weighted-only | -3.618 (-4.377, -2.859) | 9.14E-21 |
| TCM syndrome score | MD (lower=better) | A vs D | IPTW(trim), doubly robust | -1.154 (-1.981, -0.327) | 0.006222 |
| TCM syndrome score | MD (lower=better) | B vs D | IPTW(trim), doubly robust | -0.088 (-0.912, 0.735) | 0.833177 |
| TCM syndrome score | MD (lower=better) | C vs D | IPTW(trim), doubly robust | -3.626 (-4.439, -2.814) | 2.21E-18 |
| TCM syndrome score | MD (lower=better) | A vs D | Overlap weights, weighted-only | -1.133 (-1.904, -0.362) | 0.003973 |
| TCM syndrome score | MD (lower=better) | B vs D | Overlap weights, weighted-only | 0.015 (-0.792, 0.821) | 0.97157 |
| TCM syndrome score | MD (lower=better) | C vs D | Overlap weights, weighted-only | -3.579 (-4.352, -2.805) | 1.18E-19 |
| TCM syndrome score | MD (lower=better) | A vs D | Overlap weights, doubly robust | -1.123 (-1.943, -0.303) | 0.007251 |
| TCM syndrome score | MD (lower=better) | B vs D | Overlap weights, doubly robust | -0.015 (-0.846, 0.816) | 0.971676 |
| TCM syndrome score | MD (lower=better) | C vs D | Overlap weights, doubly robust | -3.583 (-4.405, -2.761) | 1.31E-17 |

Table S5. Sensitivity analyses for POP-Q outcomes (Groups A–C vs Group D).

| Outcome | Contrast | Method | Effect (95% CI) | P |
| --- | --- | --- | --- | --- |
| POP-Q stage 2 vs 1 | A vs D | Adjusted logistic (unweighted) | 0.328 (0.046, 2.341) | 0.266288 |
| POP-Q stage 2 vs 1 | B vs D | Adjusted logistic (unweighted) | 0.411 (0.083, 2.038) | 0.276445 |
| POP-Q stage 2 vs 1 | C vs D | Adjusted logistic (unweighted) | 0.283 (0.046, 1.752) | 0.174897 |
| POP-Q stage 2 vs 1 | A vs D | IPTW(trim), weighted-only | 0.975 (0.268, 3.541) | 0.969229 |
| POP-Q stage 2 vs 1 | B vs D | IPTW(trim), weighted-only | 0.572 (0.131, 2.505) | 0.45843 |
| POP-Q stage 2 vs 1 | C vs D | IPTW(trim), weighted-only | 0.260 (0.039, 1.749) | 0.165847 |
| POP-Q stage 2 vs 1 | A vs D | IPTW(trim), doubly robust | 0.946 (0.190, 4.704) | 0.946348 |
| POP-Q stage 2 vs 1 | B vs D | IPTW(trim), doubly robust | 0.652 (0.105, 4.054) | 0.646338 |
| POP-Q stage 2 vs 1 | C vs D | IPTW(trim), doubly robust | 0.325 (0.037, 2.880) | 0.312304 |
| POP-Q stage 2 vs 1 | A vs D | Overlap weights, weighted-only | 0.815 (0.191, 3.479) | 0.782098 |
| POP-Q stage 2 vs 1 | B vs D | Overlap weights, weighted-only | 0.452 (0.083, 2.460) | 0.358184 |
| POP-Q stage 2 vs 1 | C vs D | Overlap weights, weighted-only | 0.263 (0.035, 1.956) | 0.192178 |
| POP-Q stage 2 vs 1 | A vs D | Overlap weights, doubly robust | 0.766 (0.131, 4.467) | 0.766983 |
| POP-Q stage 2 vs 1 | B vs D | Overlap weights, doubly robust | 0.555 (0.069, 4.451) | 0.579253 |
| POP-Q stage 2 vs 1 | C vs D | Overlap weights, doubly robust | 0.345 (0.033, 3.566) | 0.371516 |

Table S6 Within-group comparisons of efficacy before and after treatment

| Outcome | Group | Effect measure | Adjusted estimate (95% CI) | P value |
| --- | --- | --- | --- | --- |
| Vaginal dynamic pressure | A | MD | -16.283 (-17.851 to -14.715) | <0.00001 |
|  | B | MD | -17.660 (-19.228 to -16.092) | <0.00001 |
|  | C | MD | -21.000 (-22.568 to -19.432) | <0.00001 |
|  | D | MD | -19.925 (-21.493 to -18.356) | <0.00001 |
| BSD | A | MD | -0.419 (-0.510 to -0.328) | <0.00001 |
|  | B | MD | -0.443 (-0.535 to -0.352) | <0.00001 |
|  | C | MD | -0.725 (-0.816 to -0.633) | <0.00001 |
|  | D | MD | -0.626 (-0.718 to -0.535) | <0.00001 |
| ICIQ-UI-SF score | A | RR | 1.524 (1.393 to 1.668) | <0.00001 |
|  | B | RR | 1.521 (1.391 to 1.662) | <0.00001 |
|  | C | RR | 2.526 (2.179 to 2.928) | <0.00001 |
|  | D | RR | 2.112 (1.867 to 2.389) | <0.00001 |
| POP-Q stage | A | OR | 10.444 (10.390 to 10.498) | <0.00001 |
|  | B | OR | 9.236 (9.169 to 9.303) | <0.00001 |
|  | C | OR | 21.264 (21.109 to 21.419) | <0.00001 |
|  | D | OR | 6.844 (6.794 to 6.894) | <0.00001 |
| TCM syndrome score | A | MD | 8.132 (7.646 to 8.619) | <0.00001 |
|  | B | MD | 6.943 (6.457 to 7.430) | <0.00001 |
|  | C | MD | 10.528 (10.042 to 11.015) | <0.00001 |
|  | D | MD | 7.170 (6.683 to 7.656) | <0.00001 |

Abbreviations: MD, mean difference; RR, response ratio; OR, odds ratio; BSD, bladder neck–symphysis distance; ICIQ-UI-SF, International Consultation on Incontinence Questionnaire-Urinary Incontinence Short Form.

Note: For continuous outcomes, estimates are model-based adjusted changes with approximate 95% confidence intervals calculated as estimate ± 1.96 × standard error. For ICIQ-UI-SF, estimates are response ratios with 95% confidence intervals derived on the log scale. For POP-Q stage, estimates are odds ratios transformed from ordinal model coefficients. This table is intended to summarize within-group model-based estimates; primary inference regarding comparative effectiveness is based on the adjusted between-group comparisons shown in Table 3.

**
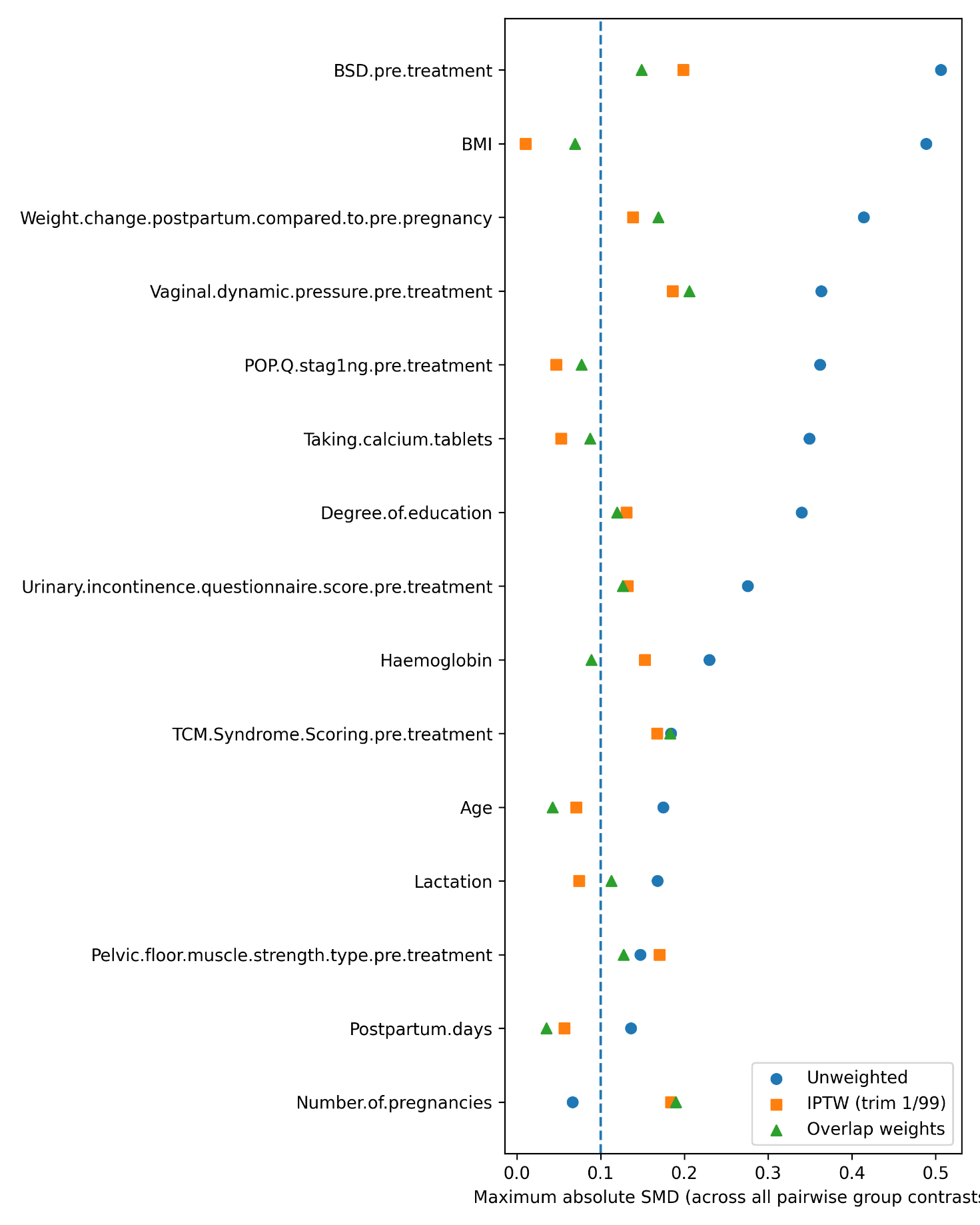
**

Figure S1 LovePlot MaxSMD

**
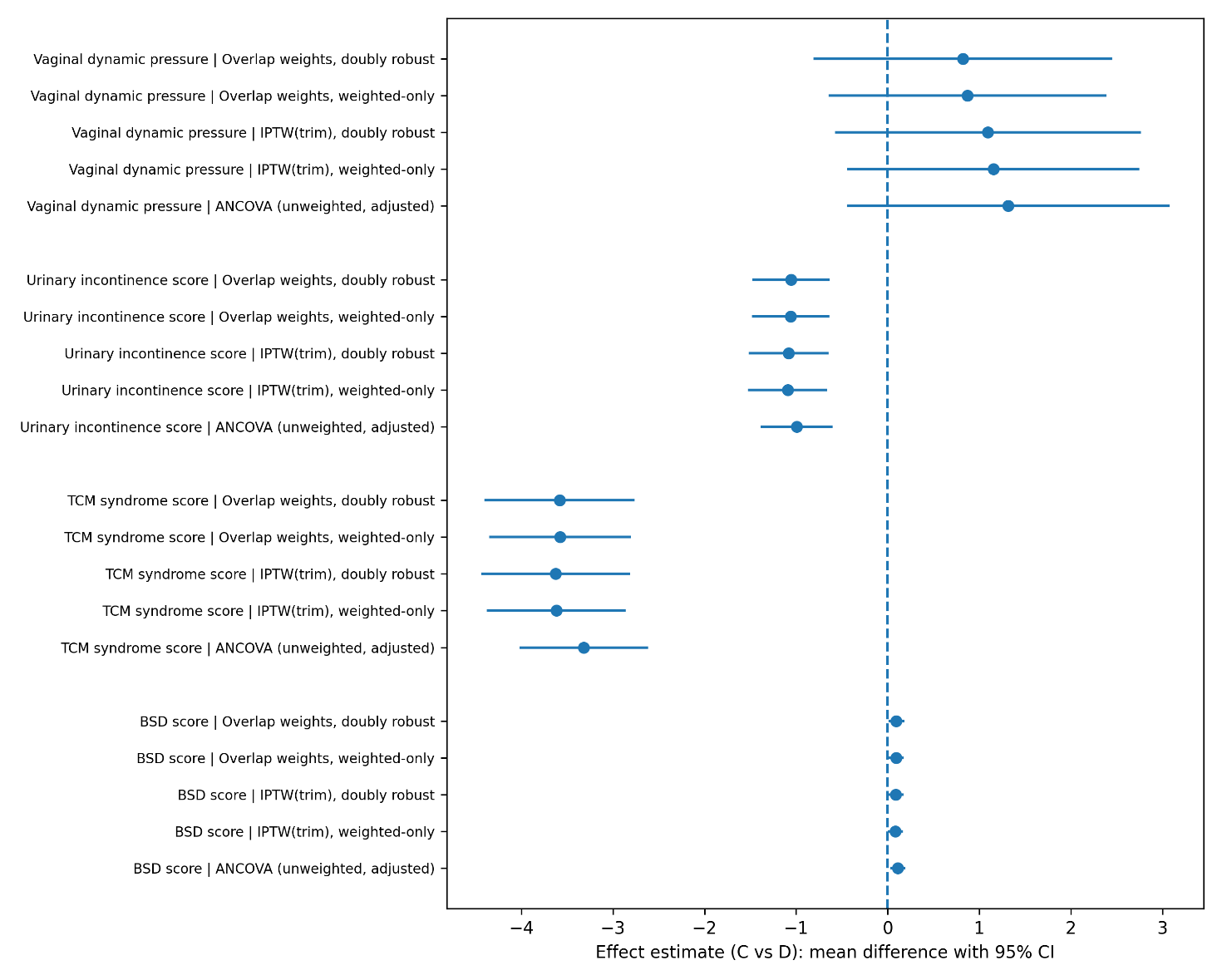
**

Figure S2. Sensitivity analyses for continuous outcomes (Group C vs D).


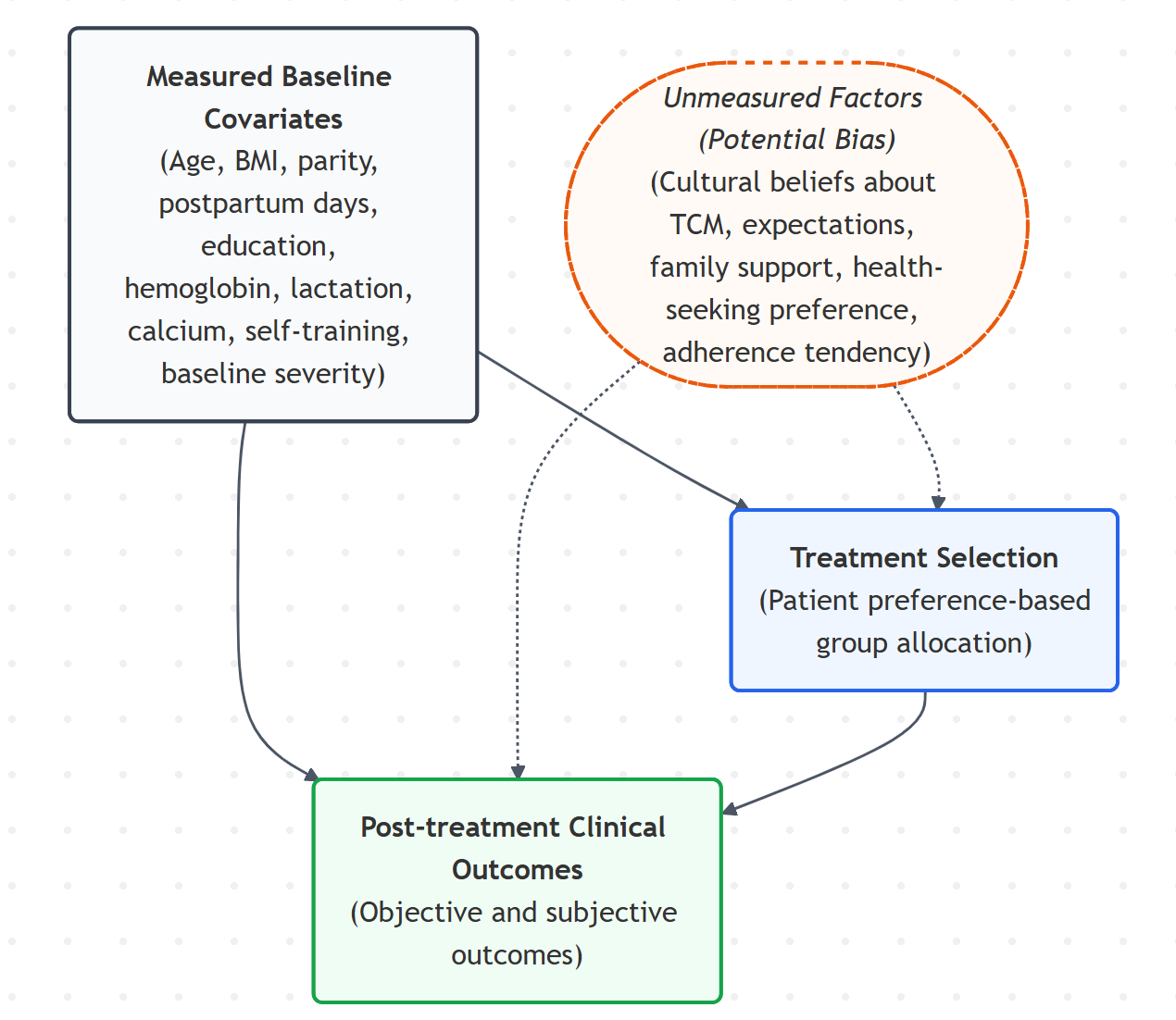


Figure S3. Directed acyclic graph illustrating the presumed confounding structure in this patient-preference cohort study.

Note: Measured baseline characteristics may influence both treatment selection and post-treatment outcomes. In addition, unmeasured factors such as prior beliefs in traditional Chinese medicine, care-seeking preferences, and adherence tendency may confound the association between treatment group and outcomes. Adjusted analyses therefore included prespecified baseline covariates, while residual confounding from unmeasured factors cannot be excluded


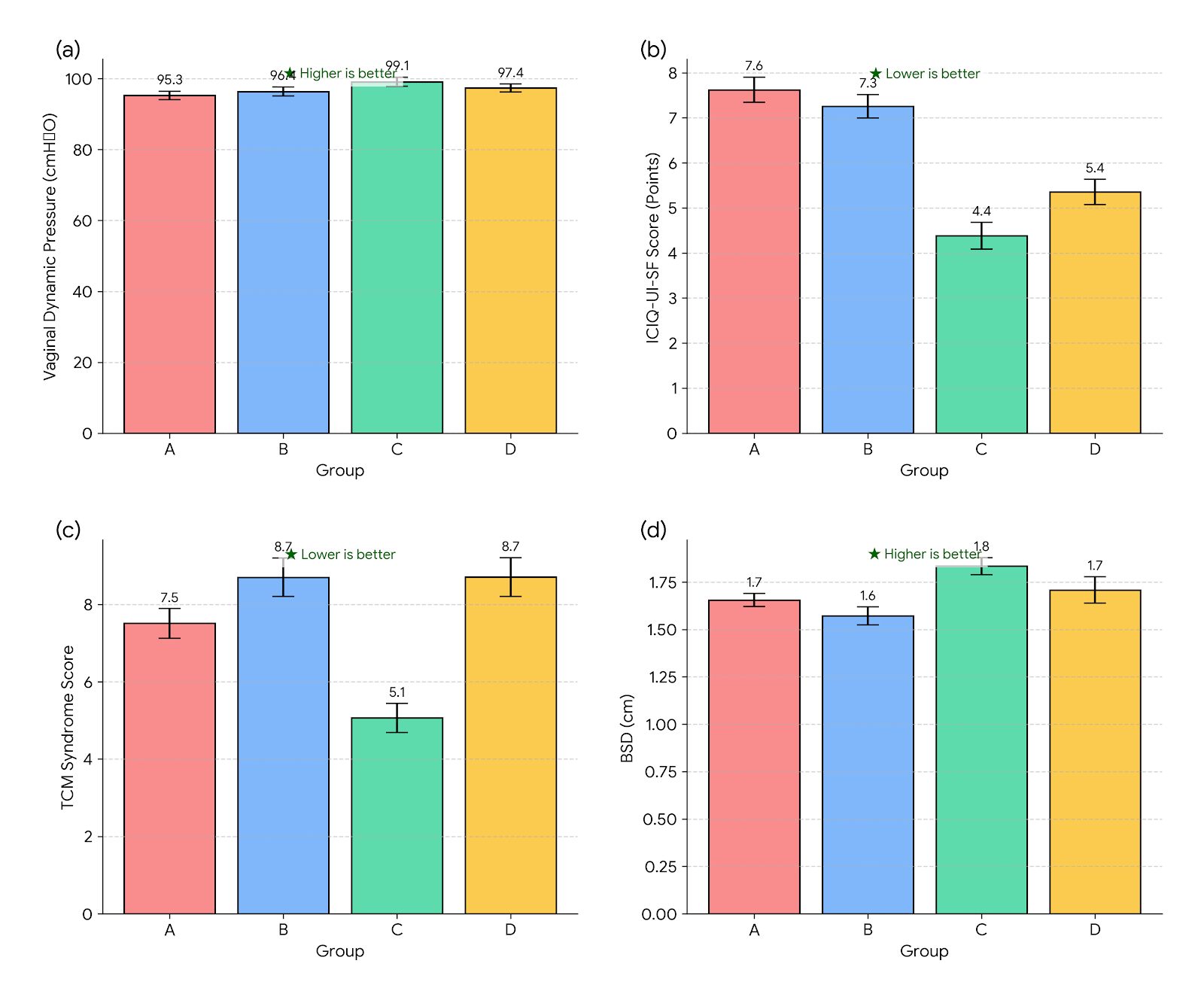


Figure S4Absolute post-treatment outcome values across groups with 95% confidence intervals.

(a) Vaginal dynamic pressure, (b) ICIQ-UI-SF score, (c) TCM syndrome score, and (d) bladder neck–symphysis distance (BSD). Bars represent observed mean values 8 weeks post-treatment, and error bars denote the 95% CIs. Directions of clinical improvement are indicated for each outcome. Absolute post-treatment outcome values across groups with 95% confidence intervals. (a) Vaginal dynamic pressure, (b) ICIQ-UI-SF score, (c) TCM syndrome score, and (d) bladder neck–symphysis distance (BSD). Bars represent observed mean values 8 weeks post-treatment, and error bars denote the 95% CIs. Directions of clinical improvement are indicated for each outcome.
